# Local Adaptation and Climate Change Vulnerability of the Relict Tree Species *Taiwania cryptomerioides* Provide Insights Into Its Conservation and Restoration

**DOI:** 10.1111/eva.70113

**Published:** 2025-05-14

**Authors:** Yang Lu, Hao Dong, Saibin Fan, Lu Yuan, Yuhui Wang, Zhuang Zhao, Yong Lai, Shixin Zhu, Jinyong Huang, Caipeng Yue, Yongpeng Ma, Ningning Zhang

**Affiliations:** ^1^ School of Life Sciences Zhengzhou University Zhengzhou China; ^2^ Henan Funiu Mountain Biological and Ecological Environment Observatory Zhengzhou University Zhengzhou China; ^3^ School of Agricultural Sciences Zhengzhou University Zhengzhou China; ^4^ Yunnan Key Laboratory for Integrative Conservation of Plant Species With Extremely Small Populations Kunming Institute of Botany, Chinese Academy of Sciences Kunming China

**Keywords:** climate change, genetic diversity, genetic vulnerability, local adaptation, relict species

## Abstract

Rapid climate change is affecting biodiversity and threatening locally adapted species. Relict species are often confined to relatively narrow, discontinuous geographic ranges and provide excellent opportunities to study local adaptation and extinction. Understanding the adaptive genetic variation and genetic vulnerability of relict species under climate change is essential for their conservation and management efforts. Here, we applied a landscape genomics approach to investigate the population genetic structure and predict adaptive capacity to climatic change for *Taiwania cryptomerioides* Hayata, a vulnerable Tertiary relict tree species in China. We used restriction site‐associated DNA sequencing on 122 individuals across 10 sampling sites. We found three genetic groups across the Chinese range of *T. cryptomerioides*: the southwest, central‐eastern, and Taiwanese groups. We detected significant signals of isolation by environment and isolation by distance, with environment playing a more important role than geography in shaping spatial genetic variation in *T. cryptomerioides*. Moreover, some outliers were related to defense and stress responses, which could reflect the genomic basis of adaptation. Gradient forest (GF) analysis revealed that precipitation‐related variables were important in driving adaptive variation in *T. cryptomerioides*. Ecological niche modeling and GF analysis revealed that the central‐eastern populations were more vulnerable to future climate change than other populations, with range contractions and high genetic offsets, suggesting these populations may be at higher risk of decline or local extinction. These findings deepen our understanding of local adaptation and vulnerability to climate change in relict tree species and will guide conservation and restoration programs for *T. cryptomerioides* in the future.

## 1 Introduction

Global climate change is profoundly affecting biodiversity and ecosystem services, potentially leading to species distribution shifts, population declines, and local extinctions (Urban 2015; Scheffers et al. 2016; Waldvogel et al. 2020). In response to climate change, species may migrate to suitable new habitats, acclimatize through phenotypic plasticity, or locally adapt to avoid extinction (Aitken et al. 2008; Capblancq et al. 2020). Local adaptation occurs when local genotypes or populations show higher fitness in their home environments compared to nonlocal genotypes or populations, driven by divergent selection resulting from environmental variation across the landscape (Aitken and Whitlock 2013; Capblancq et al. 2020). Local adaptation is thought to be common in forest tree species (Sork et al. 2016; Yang et al. 2024). Recent studies have focused on local adaptation when predicting the response of tree species to climate change (Aitken and Bemmels 2016; Dauphin et al. 2021). Therefore, understanding the mechanisms underlying local adaptation and assessing the climate change vulnerability of tree species is crucial for developing tree conservation and forest management strategies under a rapidly changing environment (Sork et al. 2013; Waldvogel et al. 2020).

Landscape genomics has increasingly been used to investigate the genomic basis of local adaptation and to estimate the vulnerability of tree species to climate change (Fitzpatrick and Keller 2015; Martins et al. 2018; Capblancq et al. 2020; Jia et al. 2020; Feng and Du 2022; Wang et al. 2023). Several methods, including genotype–environment associations (GEA) and gradient forest (GF), have recently been advocated for landscape genomics. GEA methods integrate genomic and environmental data to detect loci related to climate adaptation (Sork et al. 2013; Rellstab et al. 2015). In addition, GF models can estimate the genomic vulnerability (or genetic offset) of species and populations to future climate change, which is measured by the amount of genetic change required to track future environmental conditions (Fitzpatrick and Keller 2015; Rellstab et al. 2016, 2021). In the past few years, high‐throughput sequencing technologies, such as restriction site‐associated DNA sequencing (RAD‐seq), genotyping by sequencing, and whole‐genome sequencing (WGS), have greatly facilitated the detection of genome‐wide genetic variation and landscape genomic studies in non‐model species (Feng and Du 2022; Dauphin et al. 2023). The landscape genomics approach has been successfully used to detect adaptive genetic variation and assess vulnerability to climate change in an array of tree species, including Asian white birch ( *Betula platyphylla* ; Nocchi et al. 2023); cork oak ( *Quercus suber* ; Vanhove et al. 2021); European aspen ( *Populus tremula* ; Ingvarsson and Bernhardsson 2020); Hawaii koa ( *Acacia koa* ; Gugger et al. 2018); yellow box ( *Eucalyptus melliodora* ; Supple et al. 2018); oriental arborvitae ( *Platycladus orientalis* ; Jia et al. 2020); and lodgepole pine ( *Pinus contorta* ; Yu et al. 2022). As such, this approach offers powerful genomic tools to guide the conservation and management (such as assisted migration or assisted gene flow strategies) of forest genetic resources and to delineate seed‐sourcing strategies for restoration or reforestation projects under climate change (Hoffmann et al. 2021; Dauphin et al. 2023).

Relict tree species, for instance, ginkgo ( *Ginkgo biloba* ), dawn redwood (*Metasequoia*), and dove trees (*Davidia involucrate*) in China, have persisted through long periods of climatic change (Manchester et al. 2009; Tang et al. 2018). Most of these relict trees now occur in relatively small, isolated areas and are often restricted to local habitats (Milne and Abbott 2002; Tang et al. 2018). In particular, they are more vulnerable due to low genetic diversity resulting from genetic drift and/or limited gene exchange, compared to non‐relict species (Cao et al. 2020; Wang et al. 2023). Relict tree species are therefore excellent subjects for the study of how trees respond to climatic changes and to address questions into local adaptation and extinction (Hewitt 2000; Huang et al. 2015). Several studies have employed population or landscape genomics to investigate the genetic structure, local adaptation, and genetic vulnerability of Chinese relict tree genera and species to date, including *Cercidiphyllum japonicum* (Zhu et al. 2020); *Dipteronia* (Feng et al. 2024); *Euptelea* (Cao et al. 2020); *G. biloba* (Zhao et al. 2019); and *Pterocarya macroptera* (Wang et al. 2023). However, given the large variability of evolutionary responses to climatic change across species (Qiu et al. 2017; Zhu et al. 2020), further studies are required before we can make more general conclusions related to adaptation, evolution, and conservation of relict tree species.

In this study, we focused on investigating the potential adaptive responses of *T. cryptomerioides* Hayata (Cupressaceae) to climate change. *T. cryptomerioides* is a relict tree species with remarkable ecological and economic value in East Asia (Fu et al. 1999; Farjon 2005). The natural distribution of this species is discontinuous across mainland China, Taiwan, northern Vietnam, and northeastern Myanmar (Chou et al. 2011; Li, Chang, et al. 2018). In mainland China, the natural populations of this species are scattered in northwestern Yunnan, southeastern Guizhou, southwestern Hubei, and northeastern Fujian Provinces, and occur in mountainous areas across a wide range of elevations (500–2800 m), with diverse environmental conditions (Li et al. 2008; He et al. 2015). *T. cryptomerioides* is a large, long‐lived tree species and can reach an age of over 2000 years and a height of 80 m. This diploid (2*n* = 22), monoecious, and wind‐pollinated/dispersed conifer species is probably dependent on occasional disturbances for regeneration, and often grows in locally unstable habitats on riverbanks in deep valleys, on steep slopes, cliffs, or rocky terrains (He et al. 2015). Due to past climate change, *T. cryptomerioides* is restricted to relatively small isolated areas (Chou et al. 2011; Qin et al. 2024). However, human activities, such as deforestation and over‐exploitation, have further exacerbated its natural population decline, and it is now listed as “Vulnerable” by the IUCN (Thomas and Farjon 2011). In addition, *T. cryptomerioides* has been selected as an important tree species for reforestation in southern China and has also been introduced to arboreta and botanic gardens worldwide as an ornamental tree (Grimshaw 2011; Qin et al. 2021). Planting experiments have revealed differences in growth performance and adaptability among *T. cryptomerioides* of different provenances, showing evidence of local adaptation (Shi and Hong 1999; Wang et al. 2007; Chen et al. 2012). However, the risk of maladaptation in *T. cryptomerioides* populations locally adapted to climate may increase due to future climate change (Chiu et al. 2018; Zhao et al. 2020). The genetic diversity and structure of this species have been investigated in several previous studies using traditional genetic markers (e.g., allozymes, RAPD, ISSR, AFLP, SSRs, and chloroplast DNA fragments) (Lin et al. 1993; Ju et al. 2006; Li et al. 2008; Li, Chang, et al. 2018; Chou et al. 2011; Qin et al. 2024). However, to date, genome‐wide patterns of genetic variation remain unknown for *T. cryptomerioides*, and the impact of climate change on the adaptive response of the species has not yet been investigated.

In this study, we employed a landscape genomics approach in our investigation of the impact of climate change on the natural populations of *T. cryptomerioides* in China. We performed RAD‐seq of 122 individuals from 10 sampling sites across the Chinese distribution range of *T. cryptomerioides*. Based on single‐nucleotide polymorphisms (SNPs) derived from RAD‐seq data, our objectives were to (a) infer the species' population genetic structure, genetic diversity, and demographic history; (b) detect the role of geography and environment in shaping the spatial pattern of genetic variation and investigate the genetic basis of local adaptation in *T. cryptomerioides*; and (c) predict population vulnerability of *T. cryptomerioides* to climate change. Our investigations will provide valuable information to guide management and conservation actions for *T. cryptomerioides* under future climate conditions.

## 2 Materials and Methods

### 2.1 Sampling, RAD‐Seq Library Preparation, and Sequencing

**FIGURE 1:**
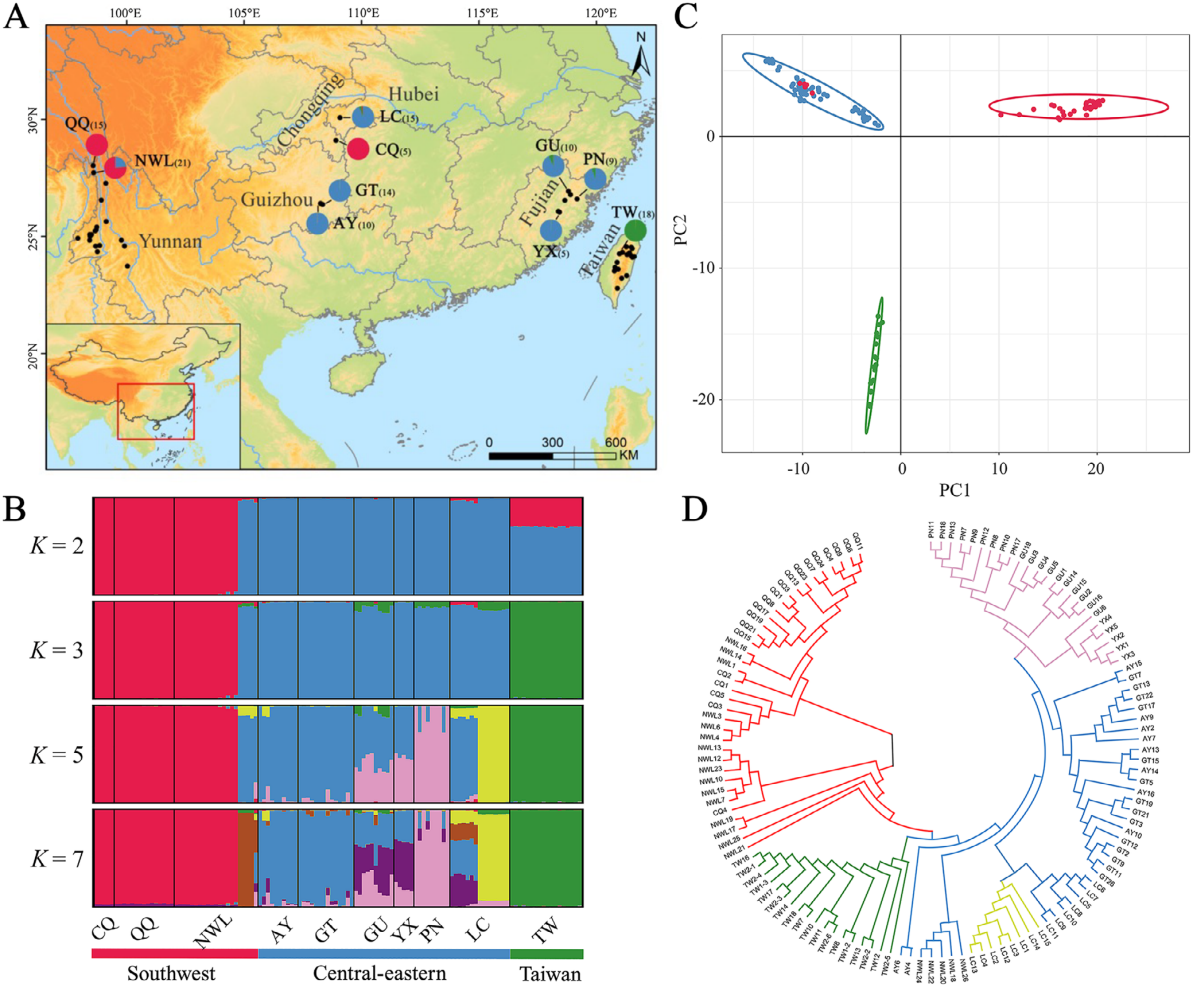
Population genetic structure of *Taiwania cryptomerioides* Hayata. (A) Locations of the 10 sampling sites in China, indicated by the pie charts. Pie charts show the ancestral composition of each sampling site with *K* = 3 inferred using STRUCTURE. The numbers in parentheses indicate the sampling sizes for each site. The black dots represent the 47 effective distribution points of *T. cryptomerioides* used in species distribution modeling. Among these points, apart from the two sampling sites in Yunnan Province and some distribution points in Taiwan, the remaining unsampled locations are relatively clear‐cut artificial plantations of *T. cryptomerioides*. (B) STRUCTURE analyses of 10 sampling sites with *K* = 2, 3, 5, 7, respectively. (C) Principal component analysis (PCA) of *T. cryptomerioides*. The colors correspond to the results of the STRUCTURE analysis with *K* = 3. (D) Neighbor‐joining (NJ) tree of the 122 sampled individuals. The branch colors correspond to the results of the STRUCTURE analysis with *K* = 5. The branches of individuals from GU and YX, which have mixed genetic components (blue and pink), were labeled as pink.

We collected samples from 10 sampling sites across the extant Chinese distribution range of *T. cryptomerioides* (Figure 1A, Figure S1), covering its primary natural populations throughout the most relevant and extant sections of its native distribution range. A total of 122 individuals were sampled, with between five and 21 mature trees sampled per sampling site (Table S1). At each site, the distance between sampled individuals was not < 20 m. Fresh leaves were collected from each sampled individual and were stored in silica gel. All plant materials were collected with the approval of the local government, and voucher specimens were deposited in the Herbarium of Zhengzhou University (ZZU). Genomic DNA from 122 individuals was extracted using a modified cetyltrimethylammonium bromide method (Doyle and Doyle 1987), and the RAD library was prepared following the protocol of Peterson et al. (2012). Genomic DNA was digested with the restriction enzymes *EcoRI* and *NlaIII*, and the digested and ligated DNA was then pooled, purified, and subjected to PCR amplification. DNA fragments ranging in size from 350 to 500 bp were selected. Library sequencing was conducted using the Illumina NovaSeq platform with 150‐bp paired‐end reads at JieRui BioScience Co. Ltd. (Guangzhou, China).

### 2.2 Processing of Illumina Data

The *de novo* pipeline in STACKS v2.54 (Rochette et al. 2019) was implemented to identify SNPs. Raw reads were demultiplexed and filtered using the procedure *process_radtags*. The remaining reads were then assembled into loci using *ustacks* with a maximum distance between stacks of *M* and a minimum read depth of *m*. The loci were clustered further into *cstacks* with mismatches allowed between samples (*n*). Following a previously described protocol (Rochette and Catchen 2017), 15 individuals were selected for a STACKS run to test whether different de novo assembly parameters in STACKS affected the number and error rates of the SNPs called. During parameter tuning, *M* = *n* was increased from 1 to 9 on the premise that *m* = 3 was guaranteed until a polymorphic RAD site could be found in 80% of samples. An optimal parameter set was then developed based on the above calculations and was used for SNP calling in all individuals in the study. Subsequently, the variant dataset was further filtered using the *populations* module in STACKS and VCFtools v4.0 (Danecek et al. 2011). After finishing de novo assembly, SNP calling and genotyping were performed using *populations*. Loci present in at least six sampling sites (*p* = 6) and in 75% of individuals in each sampling site (*r* = 0.75) were retained. We further filtered the dataset using a minor allele frequency threshold of < 0.05, the *write‐random‐snp* parameter, and retained only biallelic SNPs. SNPs with more than 40% missing data were removed using VCFtools. PGDspider v2.1.0.3 was used subsequently for file conversion to program‐specific formats (Lischer and Excoffier 2012).

### 2.3 Population Structure and Genetic Diversity

Population structure was estimated using Bayesian clustering and principal component analysis (PCA). Bayesian clustering was performed in STRUCTURE v2.3.4 (Pritchard et al. 2000). To determine the optimal number of groups (*K*), we ran STRUCTURE 10 times at *K* = 1–10. Each run was comprised of 200,000 Markov chain Monte Carlo generations following a burn‐in of 100,000 generations. The optimal *K* value was determined using the “delta‐*K*” method in STRUCTURE HARVESTER (Earl and Von Holdt 2012). Interactions of the optimal *K* value were combined and averaged by CLUMPP v1.1.2 (Jakobsson and Rosenberg 2007), and the results were visualized using DISTRUCT v1.1 (Rosenberg 2004). PCA was performed using the R package *adegenet* (Jombart 2008). Moreover, a neighbor‐joining (NJ) tree was constructed using MEGA X (Kumar et al. 2018). Based on the results of the STRUCTURE analyses, at the NWL sampling site, five individuals exhibited genetic components identical to those in the central‐eastern regions (*K* = 3). These individuals might be artificially introduced. Therefore, we removed these five individuals from subsequent analyses.

Using all the identified RAD loci, we calculated genetic diversity indices, including observed heterozygosity (*H*_O_), expected heterozygosity (*H*_E_), nucleotide diversity (*π*), inbreeding coefficient (*F*_IS_), percentage of polymorphic loci (*PPL*), and private alleles (*PA*), using the *populations* module. Pairwise genetic differentiation (*F*_ST_) values were calculated using the R v4.2.0 (R Core Team 2022) package *hierfstat* (Goudet 2005).

### 2.4 Demographic History

Based on the SNP site frequency spectrum (SFS), we inferred the demographic history of *T. cryptomerioides* using FASTSIMCOAL2 v2.7 (Excoffier et al. 2013), with *K* = 3 from the STRUCTURE analyses. Four different models were tested, mainly considering the order of lineage divergence (Figure S2). To minimize biases when determining the ancestral allelic states, we generated folded SFS using the *easySFS.py* script (https://github.com/isaacovercast/easySFS). We assumed a mutation rate of 5.9 × 10^−10^ per site per year (Fu et al. 2023) and a generation time of 150 years (Thomas and Farjon 2011). Each model was run 50 times with 500,000 coalescent simulations, as well as 50 cycles of the conditional maximization algorithm, to estimate the global maximum likelihood. The optimal model was determined according to Akaike's weight value (Excoffier et al. 2013). Additionally, we performed an analysis using the program Stairway Plot v2.0 (Liu and Fu 2020) to estimate changes in effective population sizes (*N*_e_) over time.

### 2.5 Ecological Niche Modeling

To predict the current and future distribution of *T. cryptomerioides* across China, we performed ecological niche modeling (ENM) using MaxEnt v3.4.4 (Phillips et al. 2006). To minimize the bias caused by sampling points during the model simulation process, we retained only one distribution point of *T. cryptomerioides* within each 2.5‐arcminute grid cell. Finally, a total of 47 effective occurrence records (Table S2) of *T. cryptomerioides* were obtained from the 10 sampling sites, the Chinese Virtual Herbarium (https://www.cvh.ac.cn/), and previously published studies (Li et al. 2008; Chou et al. 2011; He et al. 2015). Nineteen bioclimatic variables (Bio01–Bio19, Table S3) for the current period (1970–2000) were extracted from the WorldClim database at a resolution of 2.5 arc‐min (Hijmans et al. 2005). We also downloaded future climatic data representing moderate and high emission scenarios of the shared socioeconomic pathways, SSP245 and SSP585 (Meinshausen et al. 2020), respectively, for both the 2030s (2021–2040) and 2070s (2061–2080). This procedure was performed in R using the *raster* package (Hijmans et al. 2015). To avoid multicollinearity, six climatic variables with Pearson's correlation coefficient |*r*| < 0.8 were retained for further analyses (Figure S3), including mean diurnal range (Bio02), isothermality (Bio03), temperature seasonality (Bio04), maximum temperature of warmest month (Bio05), precipitation of driest month (Bio14), and precipitation of warmest quarter (Bio18). The model optimization and settings were as in Dong et al. (2023).

### 2.6 Detection of SNPs Under Selection

We employed BayeScan, PCAdapt, and two GEA approaches (redundancy analyses [RDAs] and latent factor mixed model [LFMM]) to identify SNPs potentially subject to selection. First, we performed BayeScan, an *F*_ST_‐based outlier detection method, to identify the outlier loci (Foll and Gaggiotti 2008). The parameters were set as follows, following the method of Li, Zhu, et al. (2018): a sample size of 5000, a thinning interval of 10, 20 pilot runs of 5000 iterations, and a burn‐in length of 50,000 iterations. Outlier loci were defined with a false discovery rate (FDR) of 0.05. For PCAdapt, outliers were identified with respect to population structure through PCA (Luu et al. 2017). Three principal components (*K* = 3) were selected. Outlier SNPs were identified under an FDR of 0.05 using the R package *qvalue* (Storey et al. 2015). Before conducting the GEA analysis, we performed a GF analysis using the R package *gradientForest* (Ellis et al. 2012) to select suitable environmental variables. Six variables (Bio2, mean diurnal range; Bio5, max temperature of warmest month; Bio9, mean temperature of driest quarter; Bio12, annual precipitation; Bio15, precipitation seasonality; and Bio17, precipitation of driest quarter) were retained according to their ranked importance (Figure S4) and pairwise correlation coefficients (|*r*| < 0.8). The multivariate RDA was then conducted using the R package *vegan* (Oksanen et al. 2019), using a standard deviation cutoff of 3 to define outliers. We then performed LFMM using the R package *LEA* (Frichot and François 2015; Gain and François 2021) to identify loci showing significant correlations with environmental gradients. Based on the results of population structure analysis, *K* = 3 was considered to be the optimal number of latent factors. The loci with *p* < 0.05 were considered to be under selection. Finally, a Venn diagram was used to show the overlap between the SNPs identified by the four methods.

In the subsequent analyses, we utilized a total of four datasets: all SNPs (based on the initial dataset of 7505 SNPs (117 individuals), we conducted a filtering process to retain only those SNPs with a missing rate of no more than 30%, resulting in a final dataset of 3349 SNPs), outlier SNPs (loci identified by BayeScan or PCAdapt methods), GEA SNPs (loci identified by RDA or LFMM methods), and putative selected SNPs (loci identified by BayeScan, PCAdapt, RDA, or LFMM methods). Additionally, by removing the putative selected SNPs from all SNPs, we constructed a neutral SNP dataset (117 individuals). Subsequently, we annotated the loci putatively under selection using BLASTx from the NCBI database with an *E*‐value ≤ 10^−5^ (Altschul et al. 1990).

### 2.7 Environmental and Geographic Contribution to Spatial Genetic Variation

In order to illustrate the role of geography and the environment in shaping the spatial patterns of genetic variation in *T. cryptomerioides* populations, we calculated isolation by distance (IBD) and isolation by environment (IBE) and then the correlation between them. The genetic distances [*F*_ST_/(1 − *F*_ST_)] were calculated using the R package *hierfstat* (Goudet 2005). The geographical distances between sites were calculated based on population coordinates (latitude and longitude) using the R package *geosphere* (Hijmans et al. 2019). The six selected climatic variables (Bio2, Bio5, Bio9, Bio12, Bio15, Bio17) were used to estimate the environmental distances between populations based on Euclidean distances with the R package *vegan*. IBD and IBE analyses were performed using Mantel tests with 999 permutations in the R package *vegan*.

We also performed RDAs in the R package *vegan*, to quantify the contribution of geography and environment to population genetic differentiation. A dependent matrix (allele frequencies for each population) and two independent matrices of environmental and geographic variables were used for the RDAs. The geographic and environmental variables were the same as those used in the above IBD and IBE analyses. A series of full and partial RDAs for different SNP sets was conducted to distinguish the independent effects of geography and environment by reciprocally constraining one of the two variables. Significance was assessed using the *ANOVA.cca* function of the R package *vegan*, with 999 permutations.

### 2.8 Genetic Offset Predictions

GF analysis was performed using the R package *gradientForest* with 500 regression trees per SNP, and all other parameters at default values. All SNPs, GEA SNPs, and the six retained climate variables were used to build the GF model and predict the genetic variation across the distribution of *T. cryptomerioides*. The ranked importance of the six climate variables was listed according to their weighted *R* ^2^ values. The split density graphs and cumulative importance curves for the six retained climate variables were presented. The GF‐predicted multidimensional genetic patterns of *T. cryptomerioides* were summarized using PCA. Following the method of Fitzpatrick and Keller (2015), the first three PCs were assigned to the RGB colors, red, green, and blue, respectively. On the diagram, similar colors represent similar expected genetic composition. We then visualized the continuous change in genomic variation of *T. cryptomerioides* throughout its range in China.

We next performed GF analyses using all SNPs and GEA SNPs to estimate the genetic offset of *T. cryptomerioides* under future climate conditions (Ellis et al. 2012; Fitzpatrick and Keller 2015). The genetic offset is a measure used to identify spatial areas where the genotype–environment relationships are most likely to be disrupted by climate change (Rellstab et al. 2021). Following the approach of Caproni et al. (2023), we confined the GF analyses to regions within China to mitigate potential biases caused by insufficient sampling of allelic diversity. We first used the current GF model to predict genetic compositions under two future scenarios (SSP126 and SSP585) during 2061–2080. We then calculated the Euclidean distances between current and future genetic compositions to serve as the metric for genetic offset (Fitzpatrick and Keller 2015). Finally, we visualized the genetic offset in geographic space, with higher offset values indicating greater vulnerability of the population in the future.

## 3 Results

### 3.1 RAD‐Seq Data Quality and Processing

RAD sequencing of the 122 *T. cryptomerioides* individuals generated a total of 346.12 Gbp data with an average of 18,913,638 raw reads per sample (Table S4). An average of 18,002,256 clean reads (range: 525,976–46,273,467; Table S4) were retained per sample after the removal of low‐quality sequences. The mean data size for samples was 2.70 Gb. The depths of coverage for samples ranged from 6.23× (AY4) to 32.36× (YX1), with a mean coverage of 9.88×. The optimal parameters were set to *M* = *n* = 4 in the STACKS analysis (Figure S5). After stringent filtering, we obtained 7505 high‐quality SNPs for subsequent analyses. The workflow diagram for the SNP datasets filtering was detailed in Figure S6.

### 3.2 Population Structure and Genetic Diversity

STRUCTURE analyses based on the 7505 SNPs identified *K* = 7 as the optimal number of genetic clusters, followed by *K* = 3 and *K* = 5 (Figure S7). At *K* = 3 (Figure 1A,B), the 10 sampling sites were divided into a southwest group (three sampling sites: CQ, QQ, NWL; red); a central‐eastern group (six sampling sites: AY, GT, GU, YX, PN, LC; blue); and a Taiwan group represented by a single population (TW; green), which were also supported by the PCA results (Figure 1C). At higher *K* values (*K* = 5, 7), the central‐eastern group could be further divided into three regional subclusters (Guizhou: AY, GT; Fujian: GU, YX, PN; and Hubei: LC), which were consistent with their geographic distributions. In addition, the NJ tree revealed a similar grouping to that generated in the STRUCTURE analyses (Figure 1D). Given that PCA distinctly separated the sampled individuals of *T. cryptomerioides* into three different groups, and considering the geographical distribution of *T. cryptomerioides*, as well as the results of STRUCTURE and the NJ tree, the investigated individuals of *T. cryptomerioides* could be roughly divided into three genetic groups (southwest, central‐eastern, and Taiwan) for the following analysis. It should be noted that five individuals from the NWL sampling site showed certain genetic components from the central‐eastern group, which may have been caused by artificial introductions, and we removed these individuals in subsequent analyses, as well as when predefined genetic group information was required. Additionally, we conducted the STRUCTURE analysis using the neutral SNP dataset (Figure S8), and the results indicated consistent genetic groups with those based on all SNPs.

The *F*_ST_ values between the three genetic groups ranged from 0.20 to 0.33, with genetic differentiation between the Taiwan and southwest groups being the highest, followed by that between Taiwan and the central‐eastern group. The *F*_ST_ value between the southwest and central‐eastern groups was the lowest (Table S5). Pairwise *F*_ST_ values between different sampling sites ranged from 0.01 (between the QQ and NWL) to 0.35 (between the CQ and TW), with high values observed between the TW and the others (Figure S9).

The *PPL* in the tested sampling sites ranged from 27.783 (CQ) to 62.693 (TW), with an average of 46.704 per site (Table 1). PAs were found in all sampling sites and ranged in number from 5 (CQ) to 665 (TW). The observed (*H*_O_) and expected heterozygosity (*H*_E_) of the sampling sites were 0.077 (YX) to 0.185 (GT) and 0.098 (CQ) to 0.204 (TW), respectively. The expected heterozygosity (*H*_E_) was higher than the observed heterozygosity (*H*_O_) in most sampling sites, with the exception of CQ and PN. The nucleotide diversity (*π*) of the sampling sites ranged from 0.110 (CQ) to 0.211 (TW). Apart from that of PN, the inbreeding coefficients (*F*_IS_) were positive in all sampling sites, indicating a degree of inbreeding. Levels of genetic diversity in the three genetic groups of *T. cryptomerioides* decreased from Taiwan (*H*_E_: 0.204 and *π*: 0.211), through the central‐eastern group (*H*_E_: 0.172 and *π*: 0.183) to the southwest group (*H*_E_: 0.105 and *π*: 0.112). Moreover, the TW group possessed the highest number of PAs (665), and the mean number of PAs was higher in the central‐eastern group (54) than in the southwest group (10). The Taiwan group also had a higher inbreeding coefficient than the other two groups.

**TABLE 1:** Summary of genetic diversity parameters for tested sampling sites of *Taiwania cryptomerioides* Hayata.

Sampling site	*PA*	*PPL*	*π*	*H*_O_	*H*_E_	*F*_IS_
Southwest group						
Chongqing (CQ)	5	27.783	0.110	0.100	0.098	0.022
Qiqi (QQ)	8	34.570	0.111	0.094	0.107	0.049
Niwaluo (NWL)^a^	17	36.240	0.114	0.084	0.109	0.084
Mean	10	32.864	0.112	0.093	0.105	0.052
Central‐eastern group						
Angying (AY)	6	57.199	0.197	0.152	0.186	0.116
Getou (GT)	57	60.492	0.202	0.185	0.194	0.049
Gutian (GU)	34	53.631	0.191	0.129	0.181	0.151
Youxi (YX)	7	29.897	0.127	0.077	0.112	0.099
Pingnan (PN)	66	46.305	0.177	0.184	0.166	−0.013
Lichuan (LC)	54	58.234	0.201	0.138	0.194	0.161
Mean	54	50.960	0.183	0.144	0.172	0.094
Taiwan group						
Taiwan (TW)	665	62.693	0.211	0.137	0.204	0.198
Total mean	102	46.704	0.164	0.128	0.155	0.092

Abbreviations: *π*, nucleotide diversity; *F*_IS_, inbreeding coefficient; *H*_E_, expected heterozygosity; *H*_O_, observed heterozygosity; PA, private alleles; PPL, percentage of polymorphic loci.

^a^ In the NWL sampling site, five individuals with the same genetic components as the central‐eastern genetic group were excluded.

### 3.3 Demographic History

**FIGURE 2:**
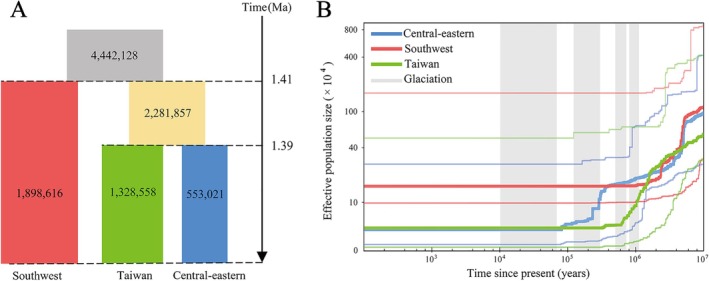
Demographic history of *Taiwania cryptomerioides*. (A) The best‐fitting demographic scenario modeled using fastsimcoal2. Each block represents a current or ancestral population with their estimated effective population size (*N*_e_). The estimated time of population divergence is indicated in million years ago (Ma). (B) Historical effective population size of the southwest, central‐eastern, and Taiwan groups over the past 10 million years, as estimated using Stairway Plot. Thick lines show the median, and thin light lines indicate the 95% pseudo‐CI defined by the 2.5% and 97.5% estimations from the site frequency spectrum (SFS) analysis. The periods of the Xixiabangma Glaciation, the Naynayxungla Glaciation, the Guxiang Glaciation, and the Baiyu Glaciation are highlighted with gray vertical bars.

From the best‐fitting fastsimcoal2 model (model 3, Akaike's weight = 1; Figure S2, Table S6), the divergence between the southwest group and the ancestral population of the Taiwan and central‐eastern groups occurred approximately 1.41 million years ago (Ma) (Figure 2A). Subsequently, the Taiwan and central‐eastern groups diverged at 1.39 Ma. The current effective population sizes of the southwest, Taiwan, and central‐eastern groups were estimated to be 1,898,616; 1,328,558; and 553,021, respectively. The ancestral population size (4,442,128) was estimated to be larger than any of these. The Stairway Plot results suggested that all groups experienced a sharp decline in population size from about 5.2 × 10^6^ years ago (Figure 2B). The southwest group has remained at a stable *N*_e_ (22 × 10^4^) since 8 × 10^5^ years ago. The Taiwan group experienced a reduction in population size until about 3.2 × 10^5^ years ago and then remained relatively stable (*N*_e_: 5.2 × 10^4^) since. The central‐eastern group experienced further contraction until about 8 × 10^4^ years ago and then maintained a low *N*_e_ of 4.7 × 10^4^.

### 3.4 Ecological Niche Modeling

The MaxEnt model of *T. cryptomerioides* had good predictive performance with a high mean test AUC (0.989 ± 0.005) (Figure S10). The predicted current distribution of *T. cryptomerioides* was basically consistent with its actual distribution (Figure S11). However, future projections exhibited significant declines in suitable habitats, particularly in the central‐eastern region (Guizhou, Hubei, and Fujian) (Figure S12). Under the most extreme climate scenario (SSP585, 2070s), the potentially suitable habitat may be reduced to approximately 41% of its current extent, and the central‐eastern populations will mostly disappear (Table S7, Figure S12). Moreover, the predicted suitable areas of *T. cryptomerioides* were mainly located in western Yunnan and central Taiwan under different climate scenarios.

### 3.5 Detection of SNPs Under Selection

BayeScan and PCAdapt identified 251 and 430 outlier loci, respectively (Figure S13, Tables S8 and S9). A total of 550 SNPs were identified as outliers using these two methods, with 131 being identified by both methods (Figure S14). RDA and LFMM were subsequently employed to identify SNPs associated with environmental variables. As a result, 126 loci were associated with at least one climatic variable, while 87 and 49 loci were identified by RDA and LFMM, respectively (Figure S14, Tables S10 and S11). There were 19, 33, 18, 22, 38, and 32 SNPs found to have significant associations with Bio2, Bio5, Bio9, Bio12, Bio15, and Bio17, respectively. Ten loci were identified as SNPs putatively under selection by RDA and LFMM (Figure S14).

We annotated all the 654 putative selected loci, and five of these were successfully annotated with a potential known function (Table 2). These genes are related to stress responses, such as the secreted RxLR effector protein 161‐like, which is involved in the pathogenicity defense response (Whisson et al. 2007; Osuna‐Caballero et al. 2024); the CBS domain‐containing protein CBSX6‐like, which responds to heat stress (Liu et al. 2021); 12‐oxophytodienoate reductase 7, which responds to drought stress (Tani et al. 2008); and the protein HOTHEAD, which responds to salt shock and regulates cuticular permeability (Chang 2016; Francom 2020). Moreover, one locus was annotated as coding for the LOB domain‐containing protein 1‐like, which is involved in secondary woody growth in *Populus* (Yordanov et al. 2010).

**TABLE 2:** Annotation information of five candidate loci under selection in this study.

Locus ID	Annotation (reference species)	*E*	% identity	Function	Reference
336	Secreted RxLR effector protein 161‐like ( *Cryptomeria japonica* )	2.86E‐15	76.744	Pathogenicity‐related protein, involved in defense response (CRK8) pathway	Whisson et al. (2007), Osuna‐Caballero et al. (2024)
586	CBS domain‐containing protein CBSX6‐like ( *Cryptomeria japonica* )	1.43E‐18	91.111	Related to anther male sterility under heat stress	Liu et al. (2021)
1393	Oxophytodienoate reductase 7 ( *Cryptomeria japonica* )	4.01E‐09	80.556	Involved in the biosynthesis of jasmonic acid, response to mechanical wounding and drought stress	Tani et al. (2008)
2599	Protein HOTHEAD ( *Cryptomeria japonica* )	7.16E‐10	96.774	Association with stress responses, respond to both salt shock and methyl jasmonate exposure; regulate cuticular permeability	Chang (2016), Francom (2020)
2808	LOB domain‐containing protein 1‐like ( *Cryptomeria japonica* )	5.01E‐08	68.182	Involved in secondary woody growth	Yordanov et al. (2010)

### 3.6 Environmental and Spatial Associations With Genetic Variation

**FIGURE 3:**
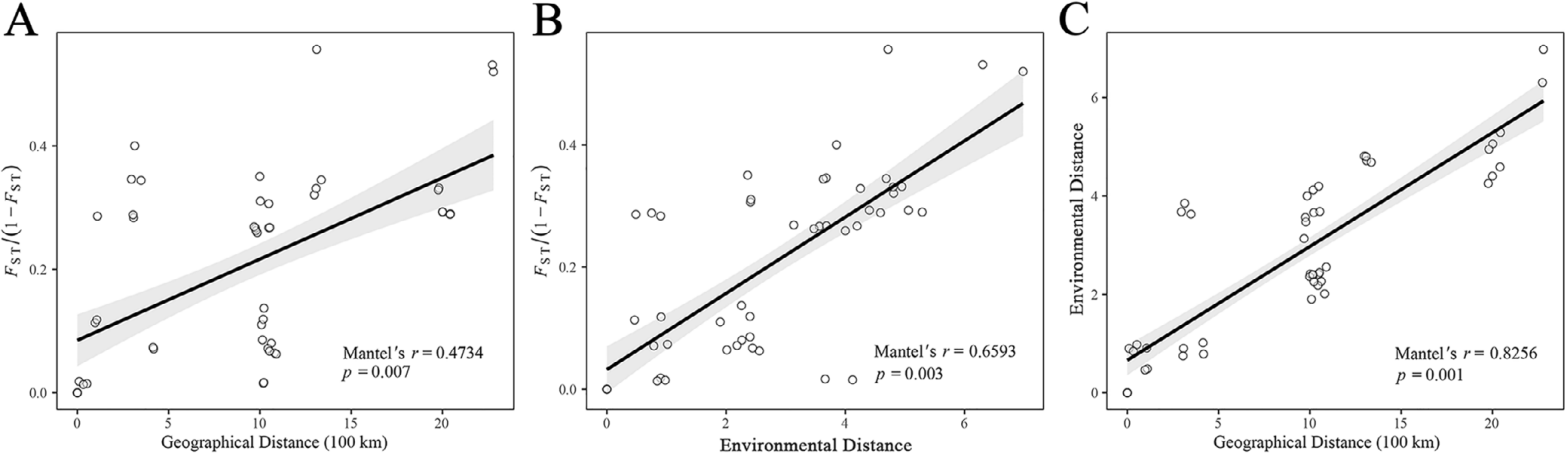
Isolation by geography and environment in *Taiwania cryptomerioides*. (A) Mantel tests of genetic distance [(*F*_ST_/(1 − *F*_ST_)] versus geographic distance and (B) environmental distance. (C) Correlation between geographic distance and environmental distance. Lines show model the predictions, and the gray shading represents the 95% confidence intervals.

We used Mantel tests to assess patterns of IBD and IBE in *T. cryptomerioides*. Significant IBD (Mantel's *r* = 0.4734, *p* = 0.007; Figure 3A) was detected. Mantel tests revealed a stronger and significant pattern of IBE (Mantel's *r* = 0.6593, *p* = 0.003; Figure 3B). The correlation between geographic distance and environmental distance was also strong (Mantel's *r* = 0.8256, *p* = 0.001; Figure 3C). We further used four datasets (all SNPs, outlier SNPs, GEA SNPs, and putative selected SNPs) to explore the environmental and geographic contribution to the spatial genetic variation in *T. cryptomerioides* (Table 3). The RDA results showed that the environment was able to explain between 36.7% and 51.3% of the total variation, while geography explained a much lower proportion of the variation, with values of 9.1% to 27%. When controlling for IBD, the environment exclusively explained higher proportions of variation (27.9% to 47.5%) than the exclusive contribution of geography (6.3% to 12%) in the four datasets. A total of 43.4% to 53% of the variation was explained by both the environment and geography, while between 47% and 56.6% of the variation remained unexplained. To explore the impact of different *K* values on the RDA, we performed PCAdapt and LFMM analyses with *K* = 5. The results showed that the SNPs obtained by the two methods were mostly overlapping under different *K* values (Figure S15), and the results of RDA were also consistent (Table S12).

**TABLE 3:** Summary of genetic variation associated with environment (env.), geography (geog.), and their combined effects based on redundancy analysis (RDA) in *Taiwania cryptomerioides*.

	All SNPs	Outlier SNPs	GEA SNPs	Putative selected SNPs
Combined fractions				
*F* ~ env.	0.367***	0.484***	0.513***	0.486***
*F* ~ geog.	0.167***	0.270***	0.091***	0.161***
Individual fractions				
*F* ~ env.| geog.	0.279***	0.308***	0.475***	0.385***
*F* ~ geog.|env.	0.095***	0.120***	0.063***	0.067***
Total explained	0.434***	0.528***	0.530***	0.503***
Total confounded	0.095	0.100	—	0.051
Total unexplained	0.566	0.472	0.470	0.497

*Note:* Data represent adjusted *R* ^2^ values, and asterisks indicate statistical significance (***p < 0.001). Total explained, total adjusted *R* ^2^ of individual fractions. Total confounded, total of individual fractions confounded between combinations of climate and geography. *F*, dependent matrix of minor allele frequencies; RDA tests are of the form: *F* ~ independent matrices|covariate matrices. env., six retained environmental variables; geo., geography (longitude + latitude).

### 3.7 GF Analysis and Genetic Offset Under Future Climate Change Scenarios

**FIGURE 4:**
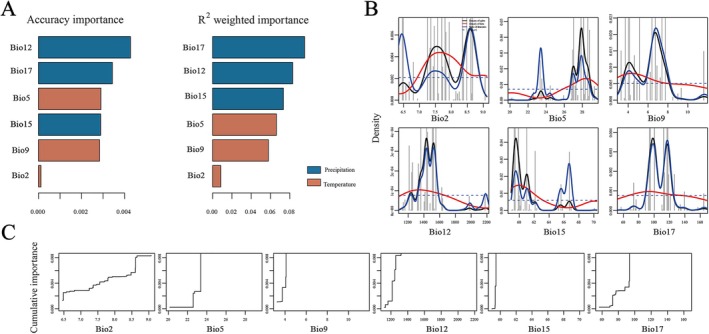
The results of gradient forest (GF) analysis based on GEA SNPs. (A) Ranked importance of environmental variables based on GF analysis. (B) Split density graph of the six environmental variables. (C) Cumulative importance of genetic variation along environmental gradients.

The GF analyses revealed significant differences in genetic composition along the distribution range of *T. cryptomerioides* and showed genetic turnover between the southwest, central‐eastern, and Taiwan regions (Figure S16). Of the six climatic variables used in the GF analysis, precipitation of driest quarter (Bio17) was identified as the most important predictor, followed by annual precipitation (Bio12) and precipitation seasonality (Bio15) (Figure 4A, Figure S16A). The max temperature of warmest month (Bio05) was the most important temperature‐related variable. The split density of the six climatic variables and cumulative allelic change along the variables is shown in Figure 4B,C, respectively.

**FIGURE 5:**
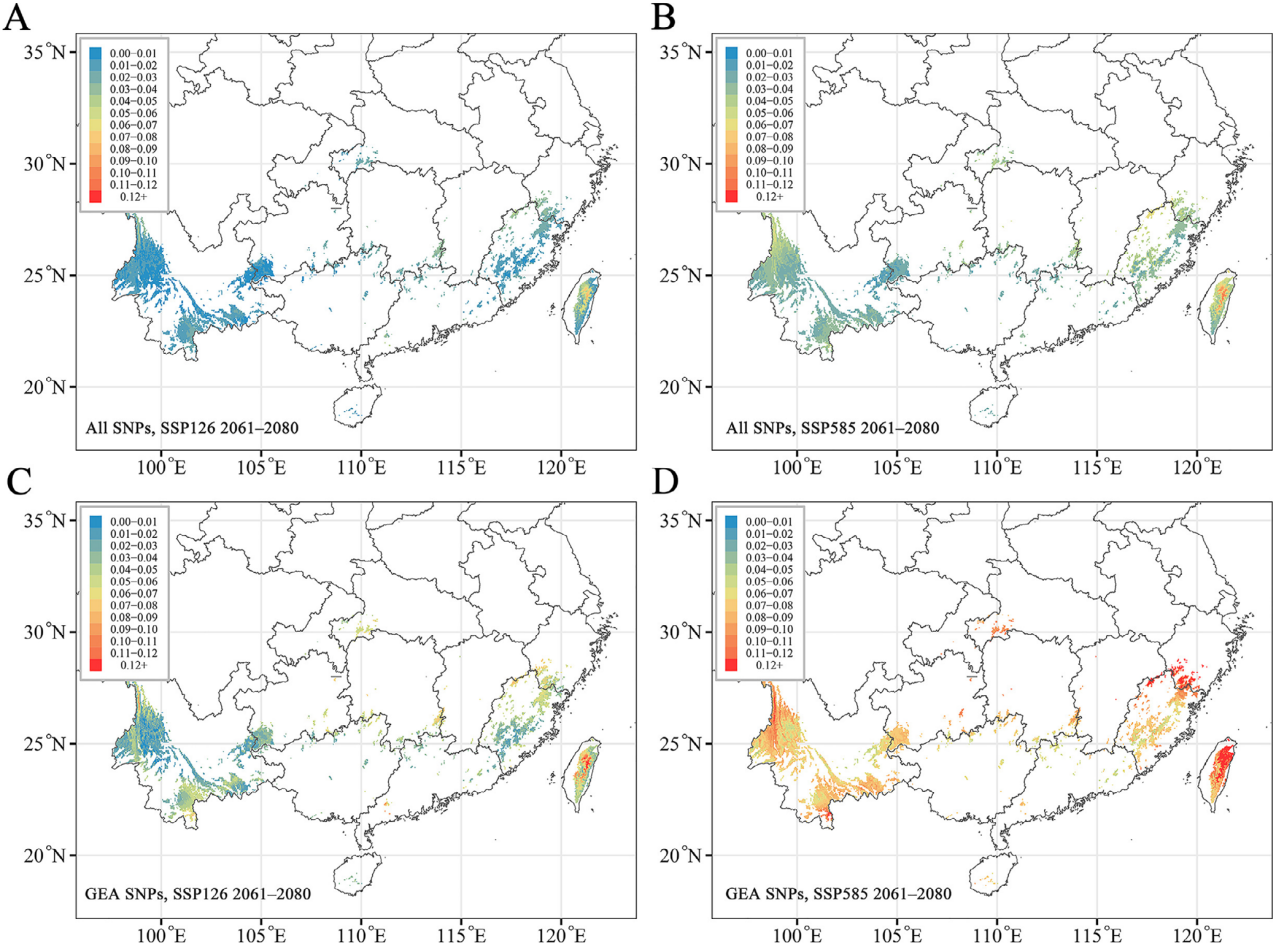
Prediction of genetic offset to future climate change based on six environment variables for (A, B) all SNPs and (C, D) GEA SNPs. (A) and (C) reflect scenario SSP126 2061–2080; (B) and (D) reflect scenario SSP585 2061–2080. The color scale represents the genomic offset.

We estimated the future genetic offset of *T. cryptomerioides* under two scenarios (SSP126 and SSP585) in the 2070s (2061–2080) based on all SNPs and GEA SNP datasets (Figure 5). The results showed that the genetic offset to climate change increased under the higher emissions scenario when compared to that under SSP126 and SSP585. In addition, the level of genetic vulnerability of the predicted regions generally increased when using the GEA SNP dataset, indicating the higher sensitivity to climate change of the GEA SNPs than all SNPs. The southwest, central‐eastern, and Taiwan regions of the *T. cryptomerioides* distribution showed genetic mismatch with different levels under both climate scenarios, indicating the genomic vulnerability of these populations to future climate change, especially for populations in Fujian Province and in the north of Taiwan Province.

## 4 Discussion

### 4.1 Population Structure and Genetic Diversity

Our population structure analysis revealed three main genetic groups in the sampled individuals of *T. cryptomerioides*: a southwest group (CQ, QQ, NWL); a central‐eastern group (AY, GT, GU, YX, PN, LC); and a Taiwan group (TW), basically corresponding to their geographic regions. Ancient geological and climatic events (i.e., glaciation cycles) are likely to have shaped the current discontinuous distribution and genetic structure of *T. cryptomerioides*. According to our fastsimcoal results, the southwest group and the ancestral population of the Taiwan and central‐eastern groups diverged approximately 1.41 Ma, and the divergence between the Taiwan and central‐eastern groups occurred at 1.39 Ma. The divergence time of the Taiwan and mainland China groups estimated in this study was later than that estimated in previous studies, that is, approximately 3.23–3.41 Ma based on cpDNA markers (Chou et al. 2011). These differences may be a result of the data sets chosen, which may reflect different aspects of evolutionary history, or the distinct mutation rates and generation times used to convert model parameters to absolute values (Xia et al. 2018; Yuan et al. 2023). According to the Stairway Plot results, *T. cryptomerioides* populations experienced a sharp decline in population size during the early Pliocene (around 5.2 Ma). The uplift of the Tibetan Plateau and the intensification of the monsoon in East Asia during the Pliocene (Wang et al. 2019; Yuan et al. 2023) likely drove population contractions in *T. cryptomerioides*. The Taiwan group then experienced a further reduction in population size in the Pleistocene, coinciding with the timing of the Xixiabangma (1.17–0.8 Ma) and Naynayxungla (0.72–0.5 Ma) Glaciations (Yang et al. 2022). Additionally, the central‐eastern group experienced a sharp population contraction during the Guxiang Glaciation (ca. 0.30–0.13 Ma). Taken together, these estimates suggest that the population demography of *T. cryptomerioides* was most likely associated with major geological events and paleoclimatic changes during the Pliocene and Pleistocene. Similar scenarios have been proposed to explain genetic structure and divergence in other East Asian tree species, such as *Platycladus orientalis* (Jia et al. 2020), *Pinus tabuliformis* (Xia et al. 2018), *Cupressus gigantean* (Yang et al. 2022), and *Quercus acutissima* (Yuan et al. 2023).

The high genetic differentiation between the three groups within *T. cryptomerioides* may be related to geographical isolation and divergent selection, as observed in other relict species in East Asia, such as *C. japonicum* (Zhu et al. 2020), *Circaeaster agrestis* (Zhang et al. 2020), and *Pterocarya macroptera* (Wang et al. 2023). The observed significant IBD pattern suggests limited gene flow between populations, while the IBE pattern highlights the role of local adaptation in shaping the population structure of *T. cryptomerioides*.

Unexpectedly, five individuals of the NWL (the southwest group) showed a close relationship with central‐eastern group populations, which was also detected in the previous study based on nSSR marker data (Qin et al. 2024). The CQ sampling site showed similar genetic components to the Yunnan populations and was distinct from its nearby populations. This suggests human‐mediated introduction of the species or long‐distance dispersal in the demographic history of *T. cryptomerioides*. Further studies are needed to better understand this phenomenon.

In this study, the results revealed a low level of genetic diversity, consistent with previous studies of this species (Li et al. 2008; Chou et al. 2011; Qin et al. 2024). Compared to studies of other endangered gymnosperms, the genetic diversity of *T. cryptomerioides* (*H*_O_ = 0.128, *H*_E_ = 0.155) was low, and was lower than that of *Cupressus gigantea* (*H*_O_ = 0.331, *H*_E_ = 0.335; Yang et al. 2022), *Pinus bungeana* (*H*_O_ = 0.275; Guo et al. 2023), and *Pseudotaxus chienii* (*H*_O_ = 0.341, *H*_E_ = 0.370; Li et al. 2021). This is in accordance with relictual characteristics, long‐term geographic isolation, and anthropogenic influences (e.g., extensive logging and habitat loss) in the evolutionary history of *T. cryptomerioides* (Thomas and Farjon 2011; He et al. 2015). In this study, inbreeding was observed within most of the studied sampling sites. The low genetic diversity and inbreeding may negatively affect species fitness and adaptation to environmental changes, and pose a risk of extinction (Zhang et al. 2020; Yang et al. 2022). The ecological consequences of this low genetic diversity in *T. cryptomerioides* would require further investigation. Interestingly, the population located in Taiwan exhibits a relatively high level of genetic diversity, with a higher number of PAs, possibly due to the presence of an ancient refugium for *T. cryptomerioides* in this region (Chou et al. 2011; Huang et al. 2015), which might have provided a comparatively stable environment for the maintenance of genetic diversity. This is also supported by our ENM analysis. Another possible explanation for the high genetic diversity of the TW population could be the result of widespread gene flow between *Taiwania* populations in Taiwan, as indicated in previous studies (Lin et al. 1993; Ju et al. 2006; Chou et al. 2011).

### 4.2 Local Adaptation

We observed that genetic distance and environmental distance were significantly correlated with each other, and environmental factors exclusively explained a much higher proportion of the genetic variation than geography in *T. cryptomerioides*. Spatial mapping of the GEAs showed clear genetic turnover between the southwest, central‐eastern, and Taiwan regions. These results reveal the signature of IBE generated by local adaptation to different environments. Moreover, a number of *T. cryptomerioides* provenance trials found differences in growth performance and adaptability among different provenances, further demonstrating local adaptation of *T. cryptomerioides* populations to their home environments (Shi and Hong 1999; Wang et al. 2007; Chen et al. 2012). The signatures of local adaptation and their effects on genetic divergence have been reported in other relict tree species from this region (Zhu et al. 2020; Wang et al. 2023). In this study, five adaptive SNPs were successfully annotated in the NCBI BLAST analysis; these five may be involved in the defense response, heat stress, drought stress, and other stress responses. Future studies, based on WGS and common garden experiments, are needed to identify more precisely the functions of those genes under selective pressure, and to elucidate the genomic signature of adaptation in *T. cryptomerioides*.

GF analysis demonstrated that precipitation‐related variables (Bio17, precipitation of driest quarter; Bio12, annual precipitation; Bio15, precipitation seasonality) were important in accounting for the adaptive variation in *T. cryptomerioides*. Similarly, precipitation‐related variables (i.e., precipitation of the driest month and precipitation of the wettest quarter) have also been identified as being the most important variables in a species distribution modeling study for *T. cryptomerioides* (Zhao et al. 2020). Chiu et al. (2018) developed a model to predict the mortality of *T. cryptomerioides* resulting from climate and other variables. They found that annual precipitation was one of the main predictor variables of tree mortality. Thus, our findings reiterate the role that precipitation factors play as key selective agents in fostering local adaptation in *T. cryptomerioides*. This is also consistent with other findings that precipitation variables play crucial roles in shaping the distributions of relict species in China (Huang et al. 2015). For example, a previous study revealed that conditions related to water were the main factors affecting seed germination, seedling growth, and survival of the relict species *Metasequoia glyptostroboides* , and that spring drought was a possible cause for the disappearance of the native *Metasequoia* from southwest China (Fan et al. 2020). Further work assessing the differential morphological and physiological responses to environmental stress (e.g., drought stress) on our study species would provide a better understanding of the adaptive mechanism of *T. cryptomerioides*.

### 4.3 Vulnerability to Climate Change

Genomic vulnerability (offset) has been used to estimate the adaptive ability of relict tree species to climate change. Genomic offset is measured by the amount of genetic change required to track future climate conditions, based on current GEAs (Cao et al. 2020; Rellstab et al. 2021; Wang et al. 2023). We found that populations of *T. cryptomerioides* in the southwest, central‐eastern, and Taiwan areas of the current distribution generally showed genomic vulnerability to climate change. However, the central‐eastern populations of *T. cryptomerioides* are smaller and more fragmented, largely as a result of frequent anthropogenic activity (Chou et al. 2011; He et al. 2015). From our ecological niche modeling results, there will be almost no suitable areas for populations of *T. cryptomerioides* in the central‐eastern region in the 2070s. Overall, the central‐eastern populations of *T. cryptomerioides* may be at higher risk of decline or local extinction than other populations. Recent studies on other species in China, such as *Euptelea pleiosperma* (Cao et al. 2020) and *Actinidia eriantha* (Zhang et al. 2023), showed a similar pattern of genomic vulnerability, revealing a higher risk of maladaptation for populations from central‐eastern China. These findings partially reflect the fact that areas of central‐eastern China should be given priority for conservation in the future. Our analysis revealed that genomic offset increased under more severe climate scenarios. Moreover, compared with all SNPs, slightly higher genomic offsets were detected for the GEA SNPs. In long‐lived forest trees with long‐generation times, such as *T. cryptomerioides*, adaptation lags are expected in response to rapid future climate change (Dauphin et al. 2021; Yuan et al. 2023). Given the low levels of standing genetic variation, our findings indicate that the persistence of *T. cryptomerioides* populations in situ seems a major challenge in the context of climate change. Nonetheless, the true evolutionary response of tree species to climate change will be more complex than the model predictions (Fitzpatrick and Keller 2015; Capblancq et al. 2020), and understanding the adaptation of *T. cryptomerioides* to future climate change will require additional studies. Our study may offer new insight into how relict species will respond to future climate challenges.

### 4.4 Management Implications

In the context of climate change, an understanding of the spatial genetic diversity and genetic vulnerability of tree species can inform conservation strategies and management decisions. Our results revealed the existence of three major genetic groups in the sampled individuals of *T. cryptomerioides*, corresponding to a southwest group, a central‐eastern group, and a Taiwan group. Our demographic simulations suggested that the three genetic groups had different demographic histories. In addition, the GF analyses revealed genetic turnover between the southwest, central‐eastern, and Taiwan regions. Hence, the three conservation units can be delineated. Our study highlights that low genetic diversity, inbreeding, and a small population size may put *T. cryptomerioides* at risk. Moreover, previous studies have reported the low natural regeneration rate and increasing rate of tree mortality of this species under climate change (He et al. 2015; Chiu et al. 2018). Thus, an integrated conservation strategy involving both in situ and ex situ approaches should be applied to maintain the existing genetic diversity and local adaptation of *T. cryptomerioides*. First, each conservation unit and extant population should be given independent in situ conservation, especially those from central‐eastern China. Although areas of central‐eastern China may appear less suitable under future climate scenarios, identifying and conserving potential refugial microenvironments within these areas are vital. These microenvironments may serve as critical microhabitats for the species' survival and should be prioritized in conservation efforts (Denney et al. 2020). Additional investigations are required to determine whether populations from central‐eastern China have already experienced negative impacts of climate change. Furthermore, assisted migration should be seriously considered for the central‐eastern populations (Aitken and Whitlock 2013). Given that the central‐eastern populations of *T. cryptomerioides* are smaller and more fragmented (Chou et al. 2011; He et al. 2015), and are in close proximity to human settlements, community participation and the enhancement of conservation awareness are also very important. Furthermore, ex situ conservation in botanical gardens, seed orchards, and seed banks should be developed; thus, effective strategies for the collection of genetically representative seeds and seedlings are essential. For tree species, populations with higher genetic diversity may have greater evolutionary potential and adaptive capacity to environmental change than less genetically diverse populations (Broadhurst et al. 2008). Thus, priority should be given to genetic conservation of the GT, LC, and TW sampling sites of *T. cryptomerioides*. Future population genomic surveys with more samples from Taiwan, northern Vietnam, and northeastern Myanmar are needed to further explore the genetic diversity and local adaptation of *T. cryptomerioides* and the threatened status of different populations.

*Taiwania cryptomerioides* is also an important tree species for reforestation and restoration projects in southern China (Qin et al. 2024). This study offers an opportunity for optimal seed zone delineation for forest restoration. Corresponding to the spatial genetic structure and conservation units, three seed zones (southwest, central‐eastern, and Taiwan) can be delineated, and the central‐eastern zone can be further subdivided into three subzones (Guizhou, Fujian, and Hubei). Our genomic‐based seed zones were partly consistent with the existing provenance trial‐based zones of *T. cryptomerioides*, which have included division of the population into three zones (Hubei, Guizhou, western Yunnan with two subzones Gongshan and Tengchong; Shi and Hong 1999); three zones (Hubei, Guizhou and Longling of Yunnan, Changning and Tengchong of Yunnan; Wang et al. 2007); and four zones (Hubei, Guizhou, Tengchong of Yunnan, Longling of Yunnan; Chen et al. 2012). Due to the lack of seed material from Fujian and Taiwan in previous provenance trials, this study provided the first genomic‐based reference for delineating seed zones over the whole distribution range of *T. cryptomerioides* in China.

Considering the predicted genetic vulnerability across the species range, we suggest conducting assisted gene flow to help high‐risk populations (e.g., the central‐eastern group and populations in the north of Taiwan) adapt to future climate conditions (Aitken and Bemmels 2016). For long‐lived tree species, mixing local seed with nonlocal seed preadapted to future environments can be effective in enhancing genetic diversity and adaptive potential (Gugger et al. 2018; Hoffmann et al. 2021). Meanwhile, common garden experiments are required for studies on adaptive trait variation and phenotypic plasticity, as assisted gene flow should be performed with caution (Capblancq et al. 2020). Overall, the results of this study improve our understanding of local adaptation and climate change vulnerability in *T. cryptomerioides*, and will guide management and conservation action for relict tree species in other regions under future climate conditions.

## Conflicts of Interest

The authors declare no conflicts of interest.

## Supporting information


**Figures** **S1–S16**

**Tables** **S1–S12**

## Data Availability

The VCF files and all scripts used in this study are available at https://github.com/luyang‐zzu/Taiwania_landscape_genomics.
