# Supplementary material for: Local Adaptation and Climate Change Vulnerability of the Relict Tree Species Taiwania cryptomerioides Provide Insights Into Its Conservation and Restoration
Source: Evol Appl. 2025 May 14;18(5):e70113. doi: 10.1111/eva.70113 (PMC12078759; doi:10.1111/eva.70113)
Supplement: Supplementary file 1 — Figures S1–S16 [file EVA-18-e70113-s002.docx]

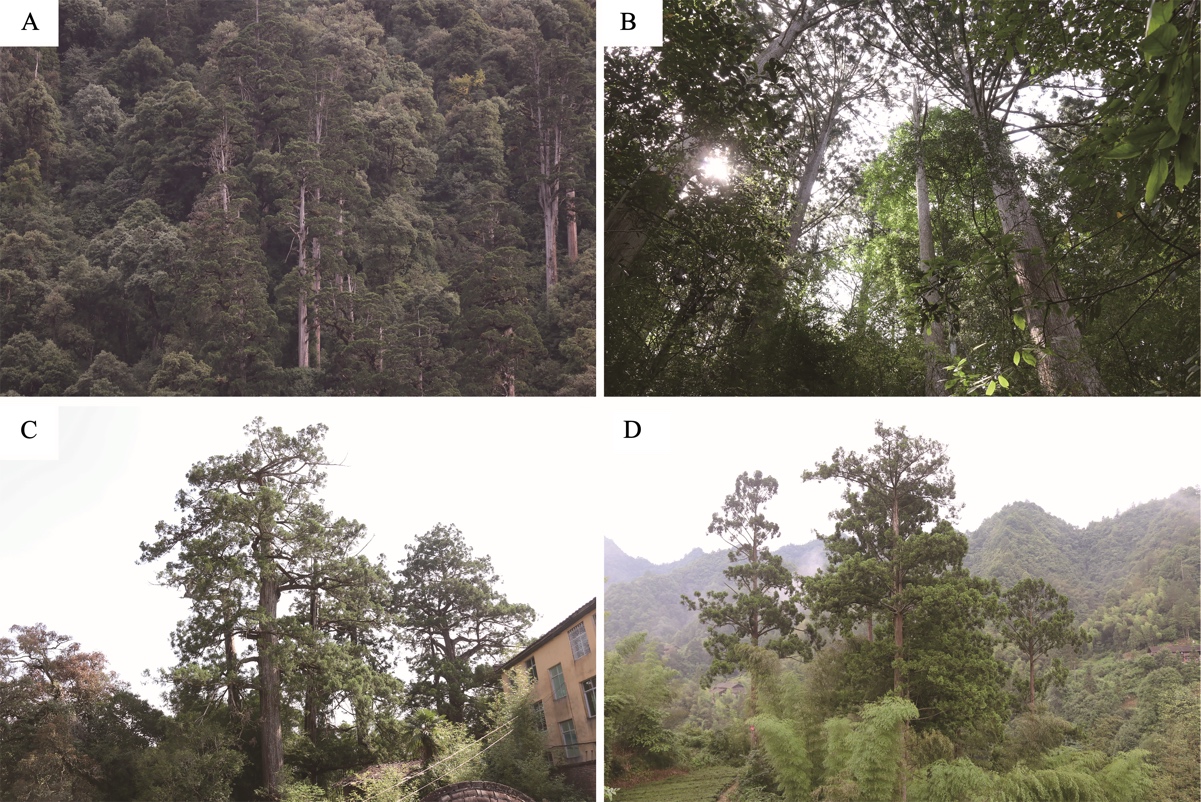


**Figure S1** The photographs show the representative populations of *Taiwania cryptomerioides* sampled in this study. (A) Qiqi (QQ), Yunnan. (B) Angying (AY), Guizhou. (C) Gutian (GU), Fujian. (D) Lichuan (LC), Hubei.


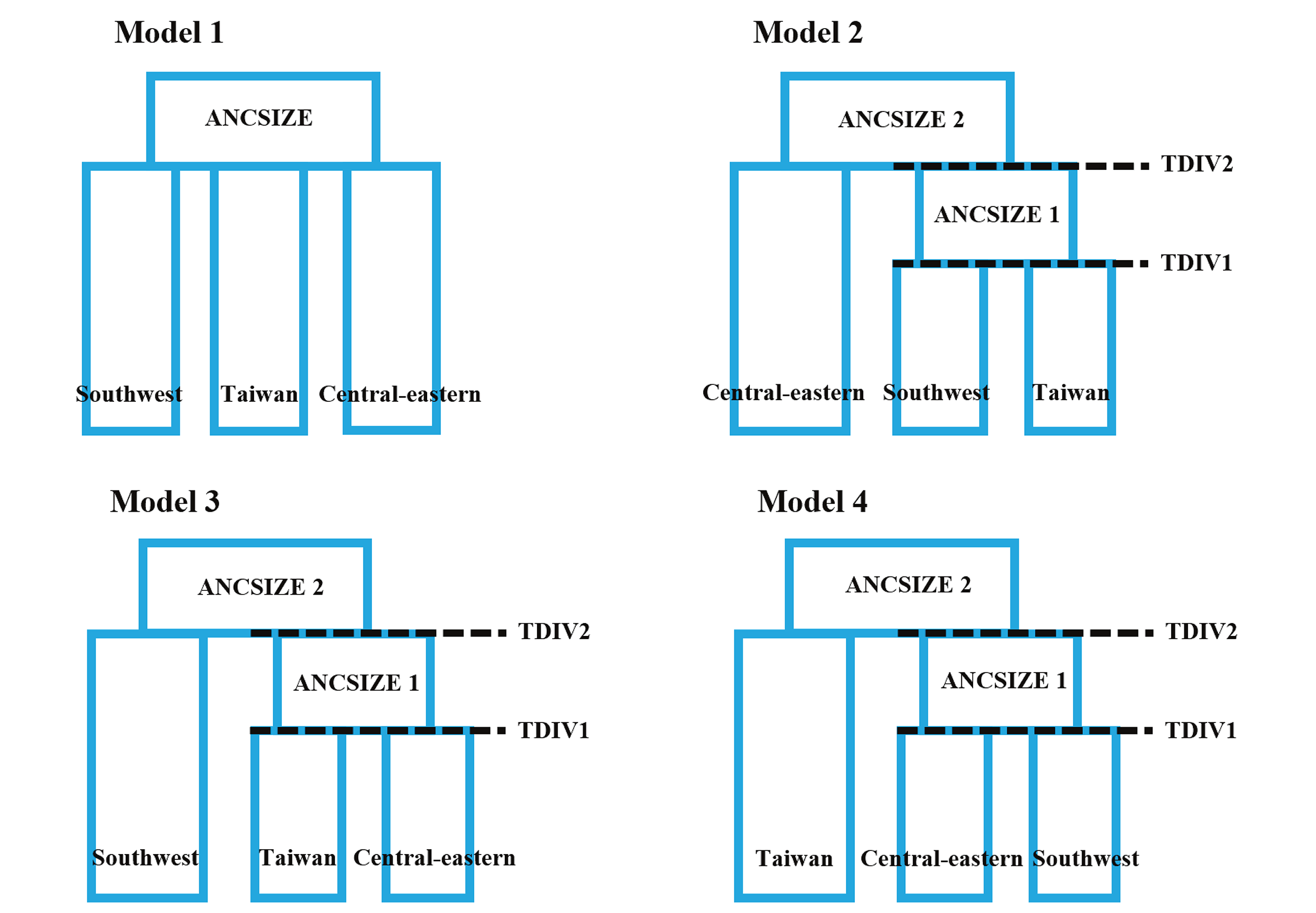


**Figure S2** Four candidate demographic models inferred from fastsimcoal2. Model 1 assumes the simultaneous divergence of the central-eastern, southwest, and Taiwan groups. Model 2 suggests that the central-eastern group first diverged from the ancestral population ANCSIZE2 at TDIV2, followed by the divergence of the southwest and Taiwan groups from ancestral population ANCSIZE1 at TDIV1. Models 3 and 4 are constructed based on the same rationale.


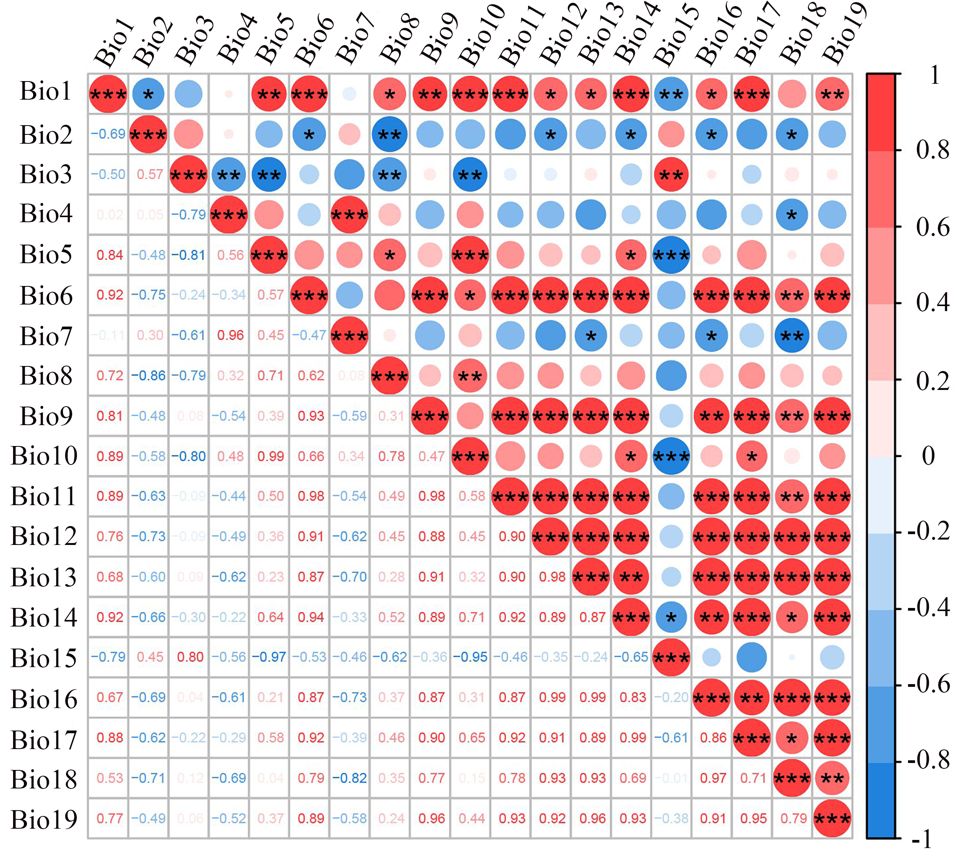


**Figure S3** Pearson’s correlation analysis of 19 environmental variables. Each grid represents the correlation coefficient between two variables, ranging from -1 to 1, where 1 indicates a perfect positive correlation, -1 indicates a perfect negative correlation, and 0 indicates no correlation. The color and size of the circles indicate the strength and direction of the correlation. The legend on the right shows the correlation coefficient corresponding to the colors, with red indicating positive correlation and blue indicating negative correlation. Significance levels: *, *p* < 0.05; **, *p* < 0.01; ***, *p* < 0.001.


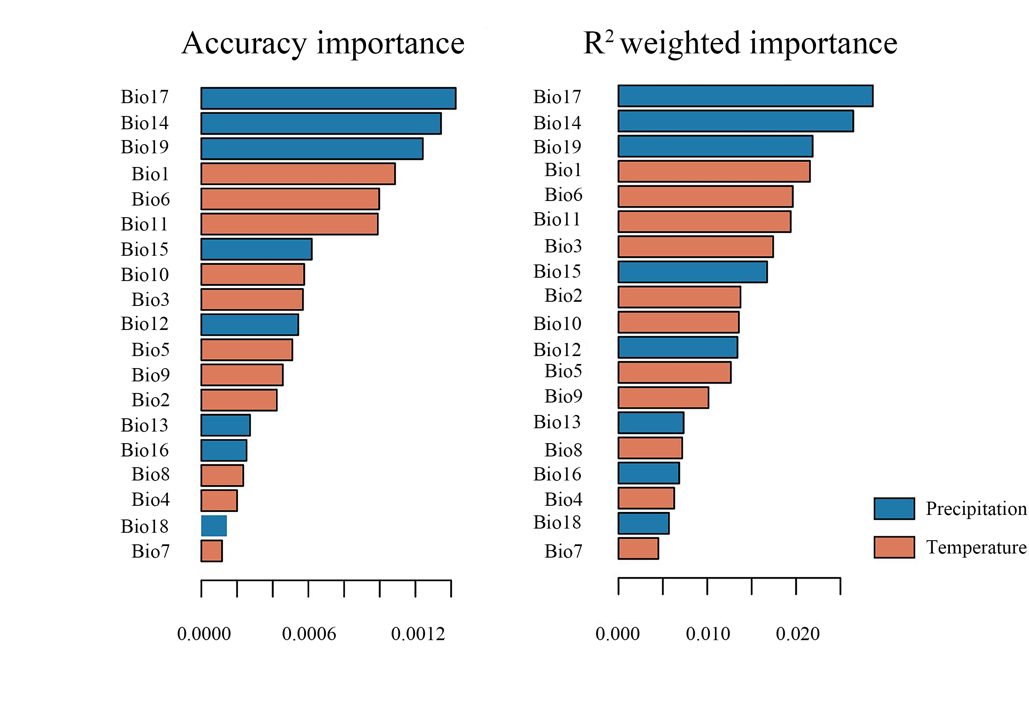


**Figure S4** Accuracy importance and R^2^-weighted importance of environmental variables based on the GF analysis. The x-axis represents the contribution of each variable to the model's predictive accuracy and to the model's explanation of variance, respectively. Blue bars represent precipitation-related variables, and orange bars represent temperature-related variables.


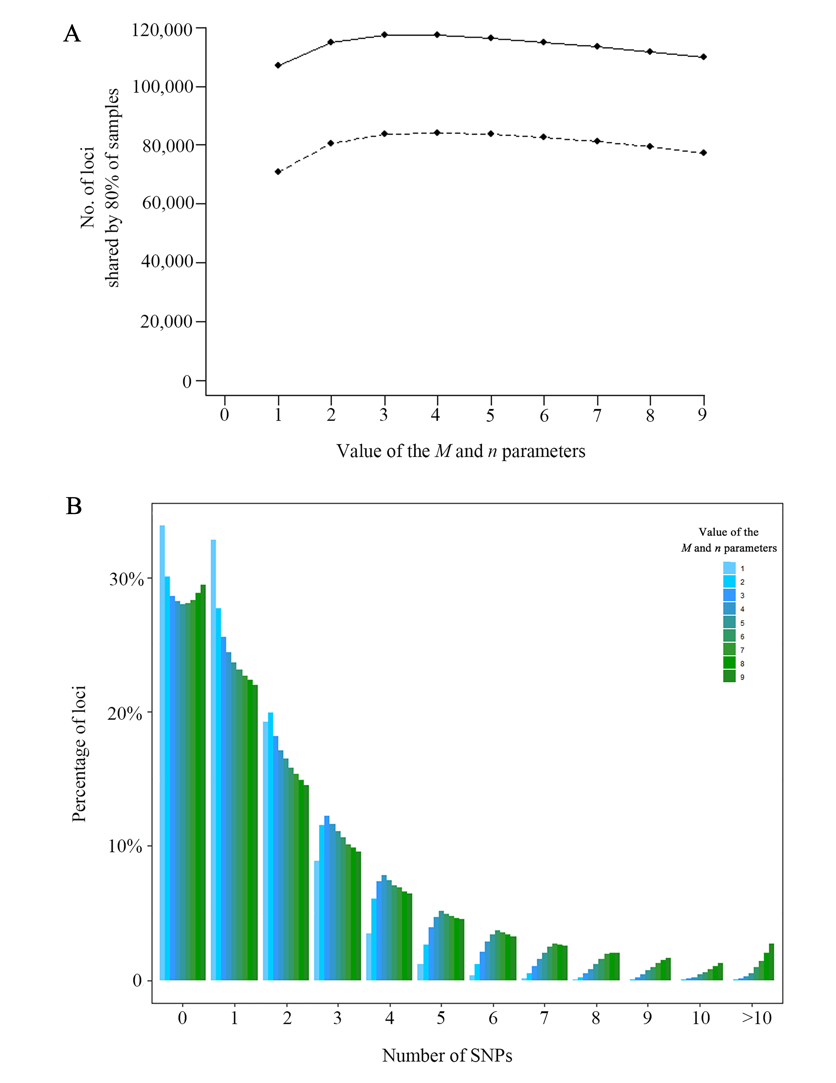


**Figure S5** Selection of *M* and *n* parameters in the STACKS analysis. (A) The number of loci shared across samples as *M* and *n* increases. All loci (solid line) and polymorphic loci (dashed line). (B) The distribution of the number of single nucleotide polymorphisms (SNPs) per locus for a range of *M* and *n* values.


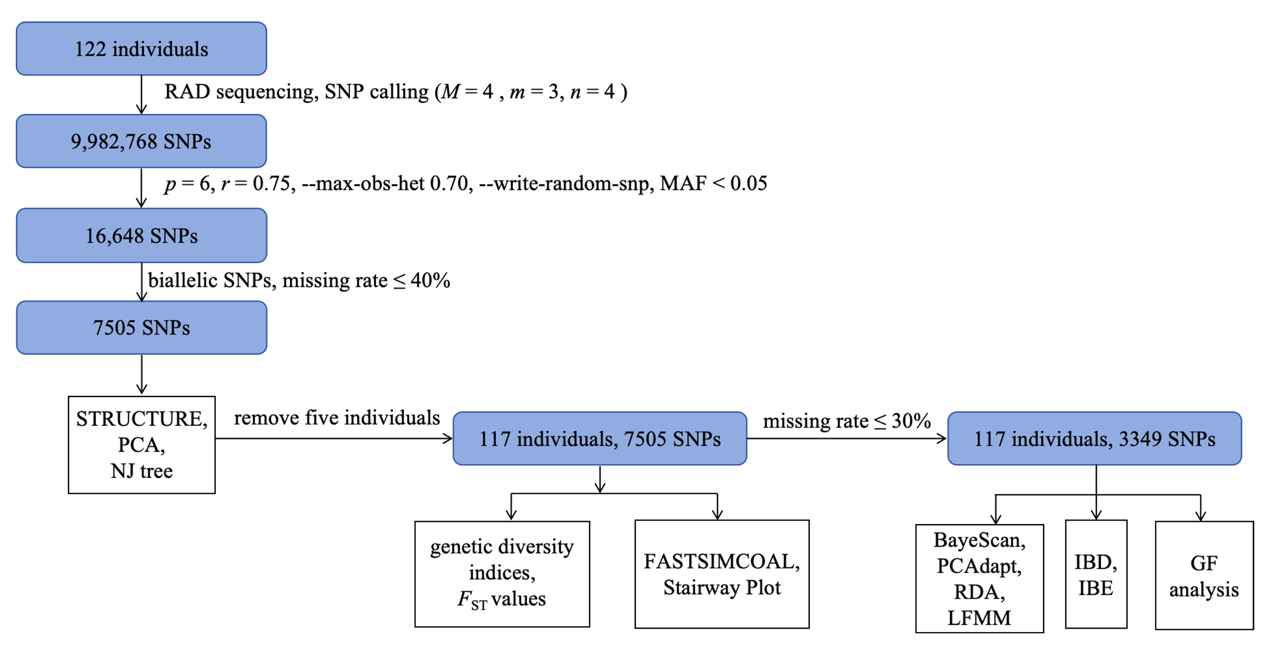


**Figure S6** Filtering workflow of single-nucleotide polymorphisms (SNPs) datasets for *Taiwania cryptomerioides* in this study.


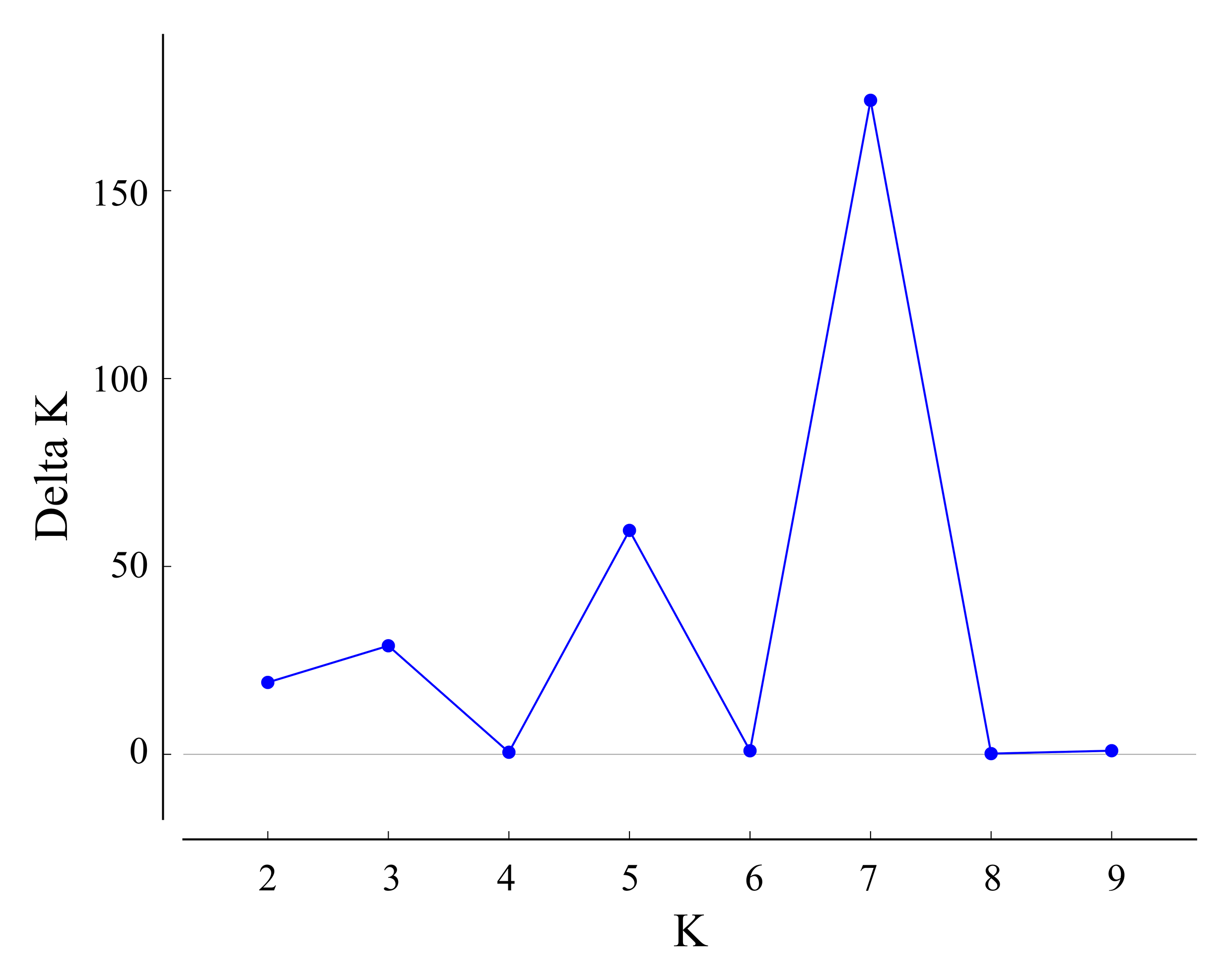


**Figure S7.** The optimal *K* value identified using the Delta*K* (△*K*) method in STRUCTURE.


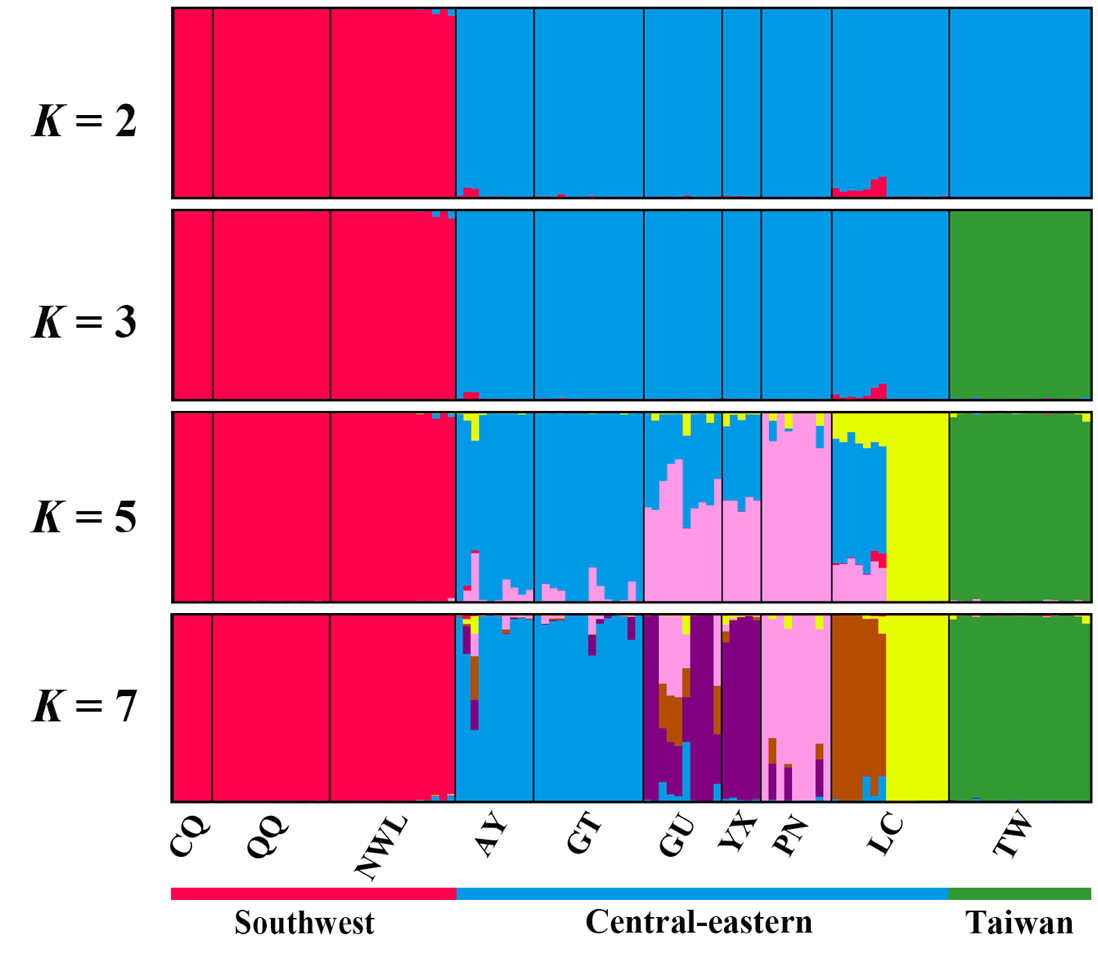


**Figure S8** STRUCTURE analyses based on the neutral SNPs dataset for *Taiwania cryptomerioides*.


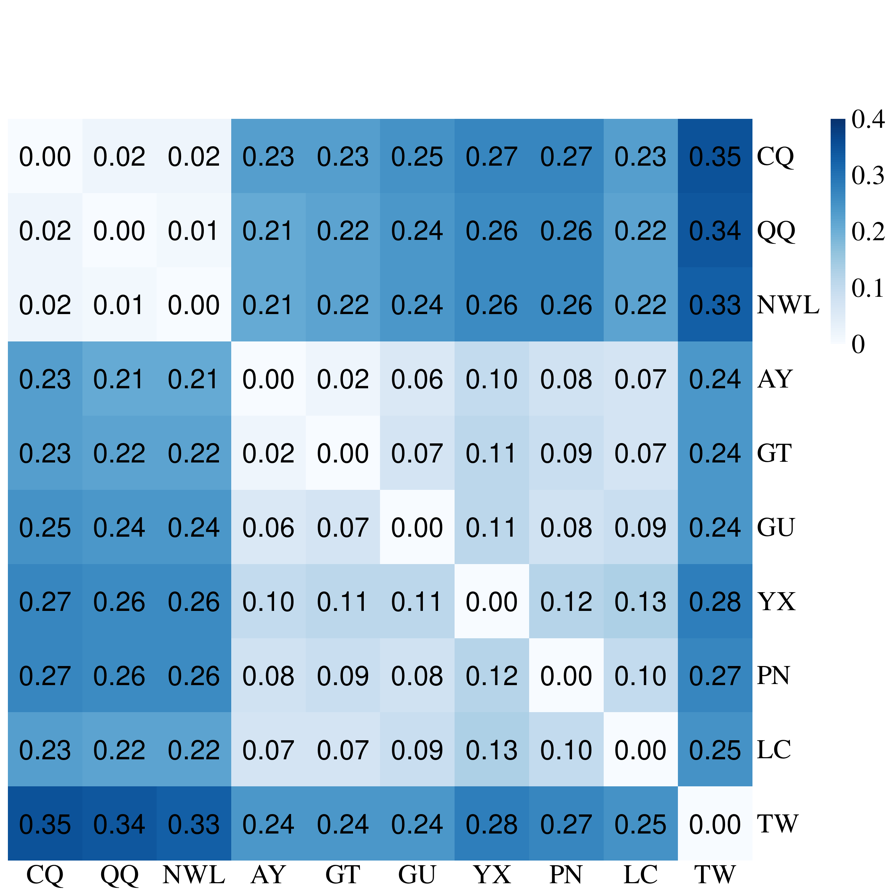


**Figure S9** Heatmap showing pairwise *F*_ST_ values among sampling sites of *Taiwania cryptomerioides*. In the NWL sampling site, five individuals with the same genetic components with the central-eastern genetic group were excluded.


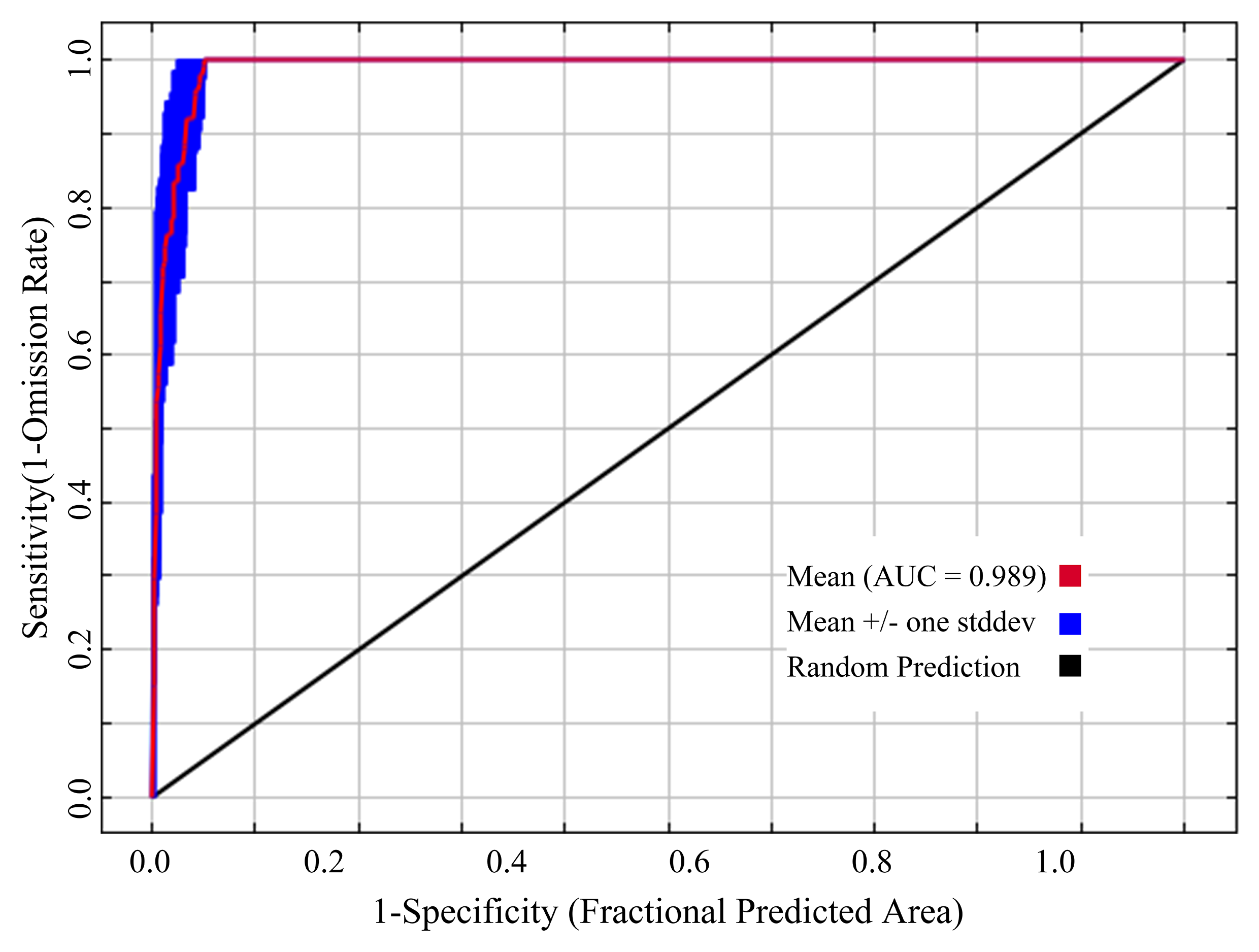


**Figure S10** The receiver operating characteristic (ROC) curve of the distribution model.


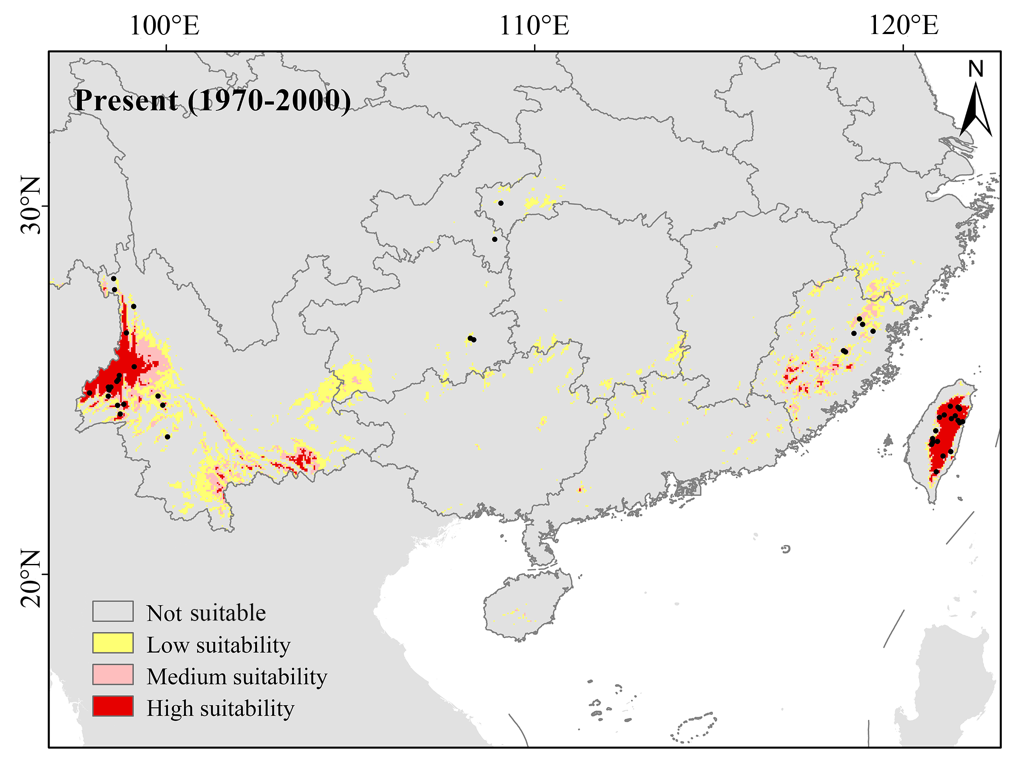


**Figure S11** The potential distribution of *Taiwania cryptomerioides* under current climatic conditions. The potential distribution was divided into four grades, including unsuitable (0.00–0.25), low suitability (0.25–0.50), medium suitability (0.50–0.75), and high suitability (0.75–1.00). The black dots indicate the locations of species distribution used in the simulation.


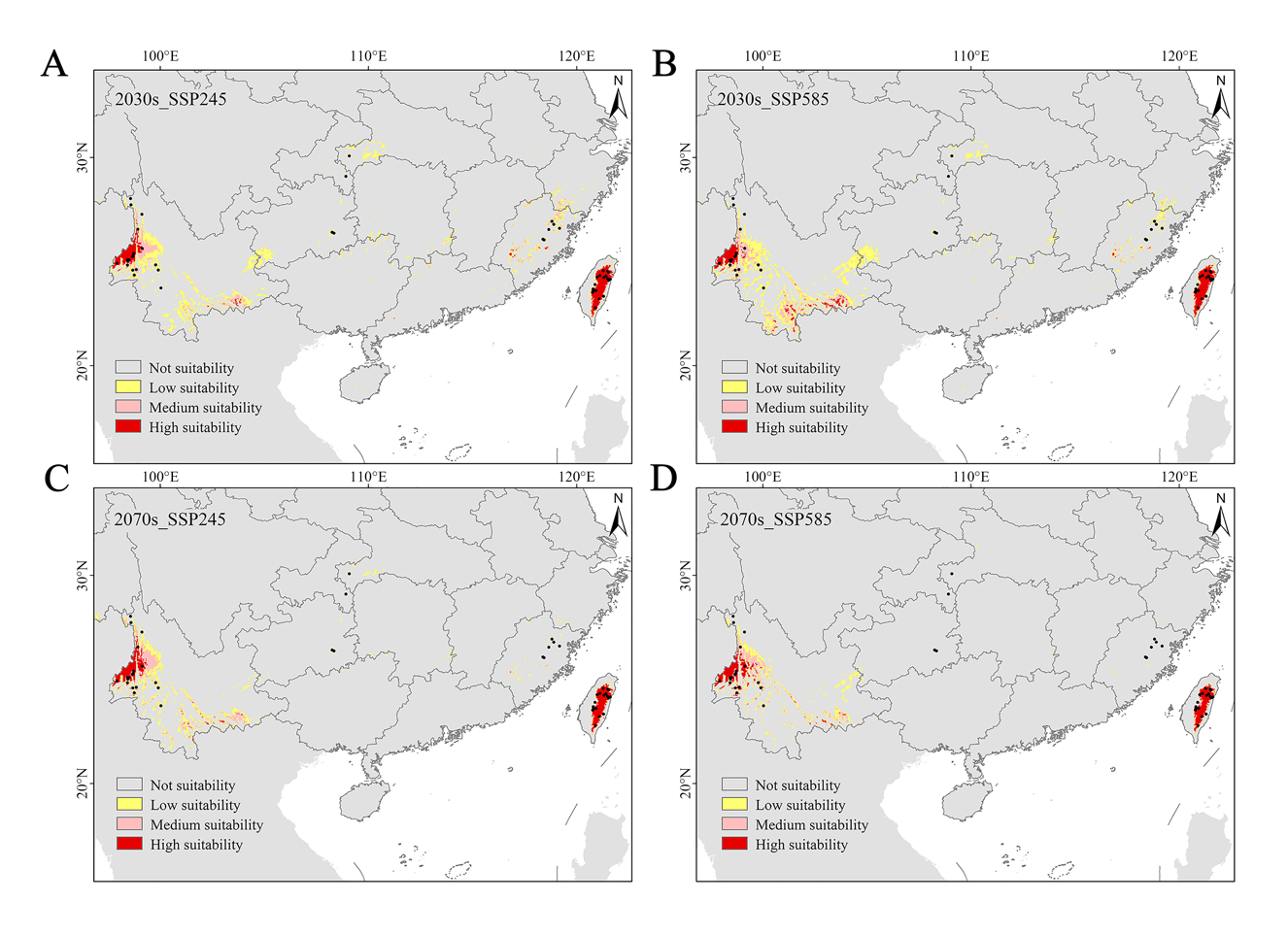


**Figure S12** The potential distribution of *Taiwania cryptomerioides* under different future climate scenarios. (A) 2030s_SSP245. (B) 2030s_SSP585. (C) 2070s_SSP245. (D) 2070s_SSP585.


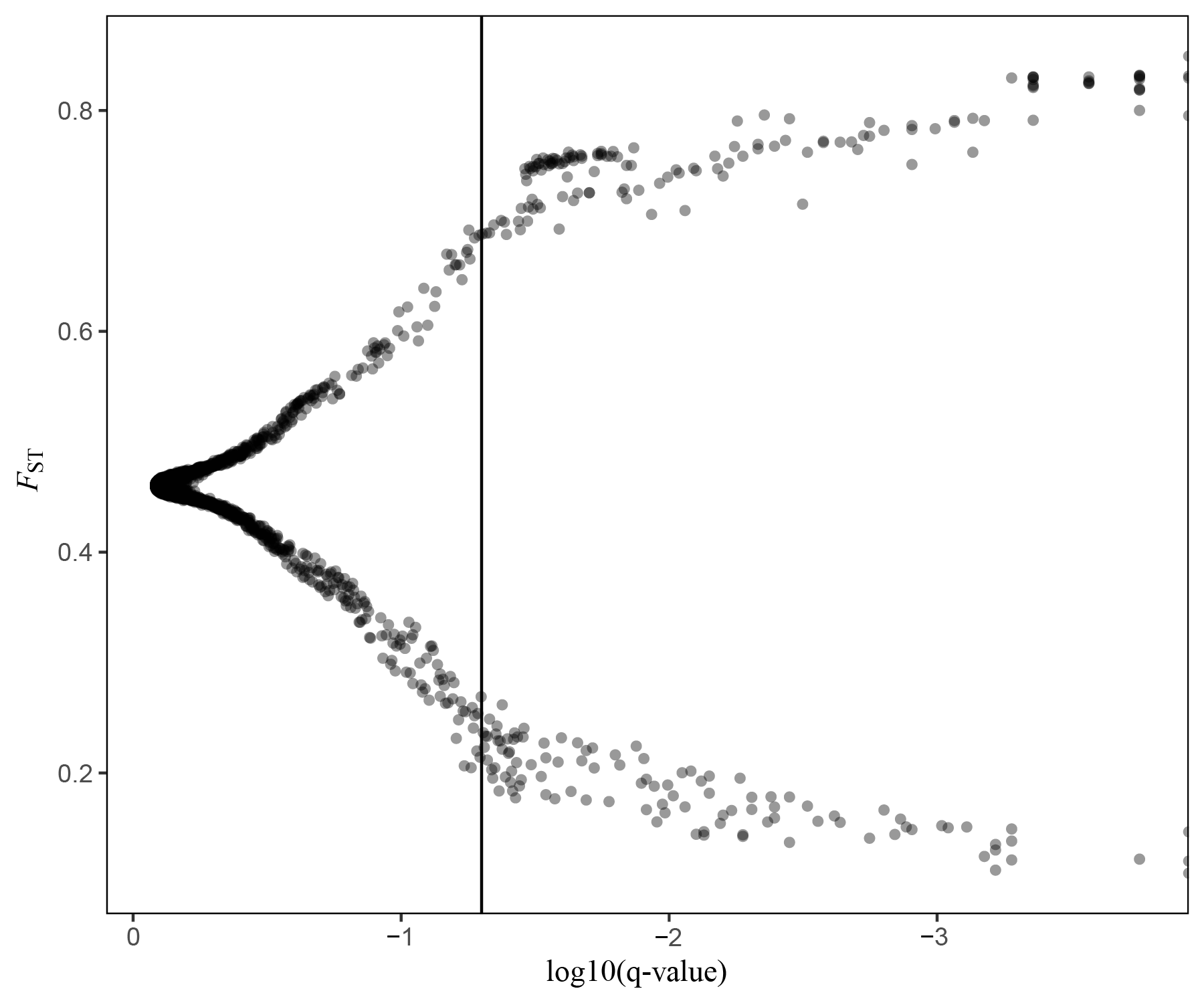


**Figure S13** *F*_ST_-outlier SNPs in *Taiwania cryptomerioides* identified with BayeScan. Each dot represents a SNP, and its position and height reflect the *F*_ST_ value of the SNP or the strength of the selection signal. SNPs located to the right of the threshold line are considered to be significant *F*_ST_ outliers.


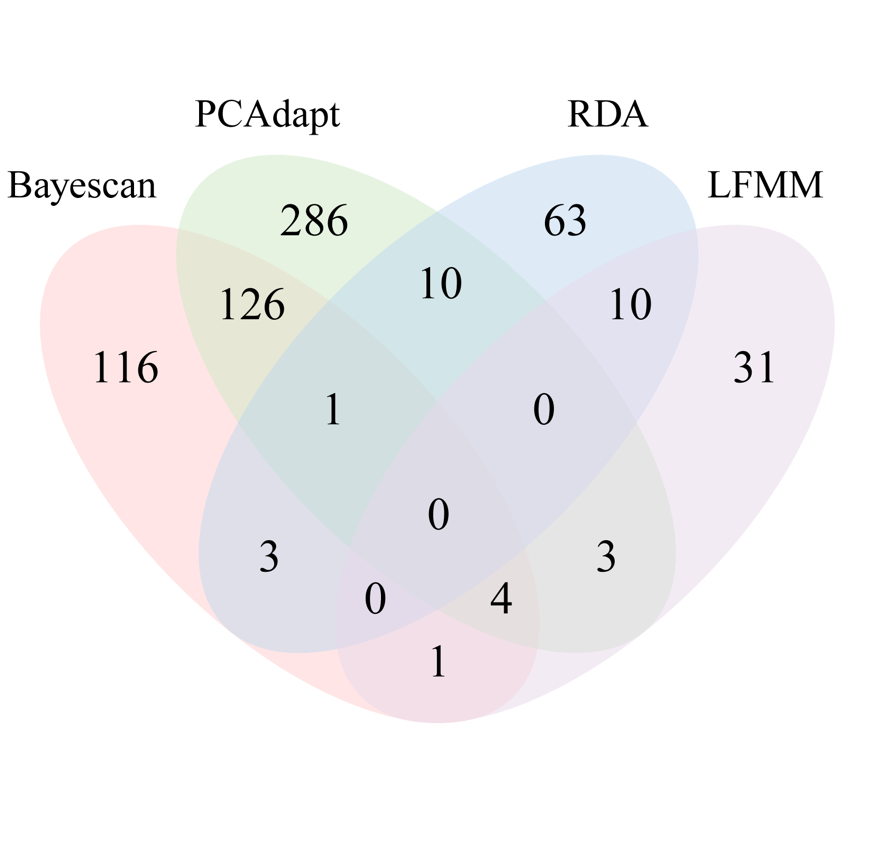


**Figure S14** Venn diagram showing overlaps between the SNPs identified using the four different methods, BayeScan, PCAdapt, RDA and LFMM.


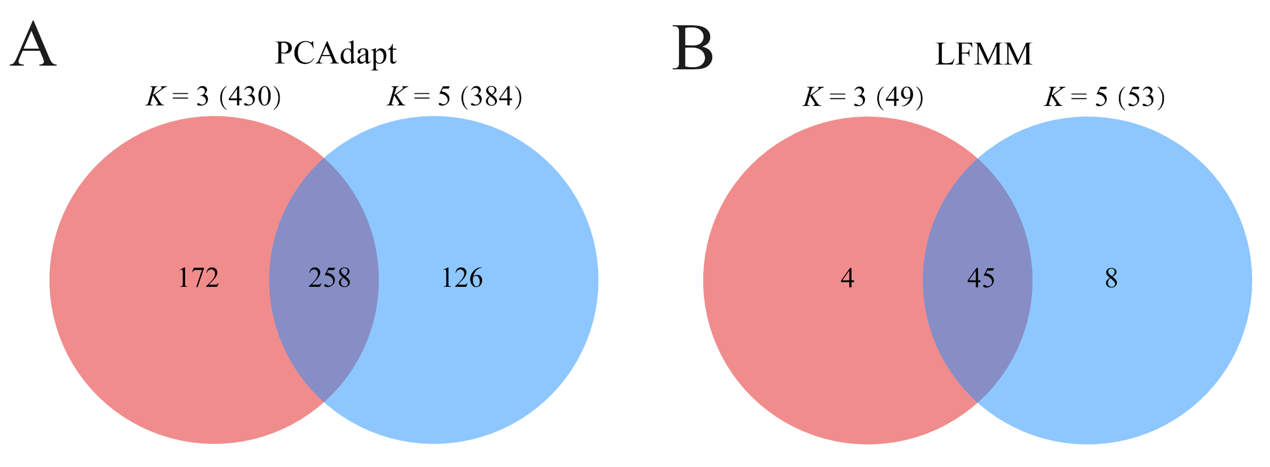


**Figure S15** (A) Venn diagram showing overlaps between the SNPs identified by PCAdapt with *K* = 3 and *K* = 5, respectively. (B) Venn diagram showing overlaps between the SNPs identified by LFMM with *K* = 3 and *K* = 5, respectively.


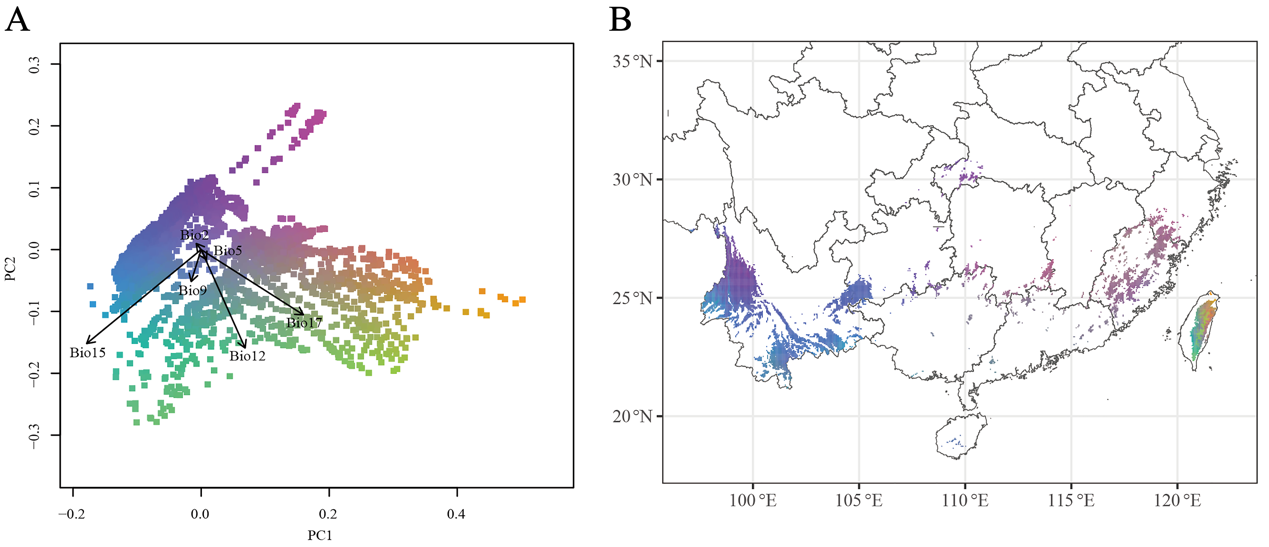


**Figure S16** Genotype–environment relationships in *Taiwania cryptomerioides* based on gradient forest (GF) analysis. (A) Principal component analysis (PCA) biplot of the GF-predicted genetic variation. Each dot represents a sample, with its position in the principal component space indicating the sample’s genetic variation. The varying colors of the dots represent distinct gradients of genetic variation as summarized by PCA. (B) GF mapped genotype–environmental associations across the *T. cryptomerioides* distribution area. Colors represent the PCA-summarized gradients in genetic turnover. The first three PCs were each assigned to a RGB color, red, green, and blue. Locations with similar colors are expected to harbor populations with similar genetic composition.
