# Supplementary material for: Local Adaptation and Climate Change Vulnerability of the Relict Tree Species Taiwania cryptomerioides Provide Insights Into Its Conservation and Restoration
Source: Evol Appl. 2025 May 14;18(5):e70113. doi: 10.1111/eva.70113 (PMC12078759; doi:10.1111/eva.70113)
Supplement: Supplementary file 2 — Tables S1–S12 [file EVA-18-e70113-s001.docx]

**Table S1** Details of geographic locations and sample size (*N*) for 10 sampling sites of *Taiwania cryptomerioides* in China.

| Population | Location | *N* | Longitude (°E) | Latitude (°N) | Altitude (m) |
| --- | --- | --- | --- | --- | --- |
| CQ | Youyang, Chongqing | 5 | 108.91 | 29.10 | 824 |
| QQ | Qiqi, Gongshan, Yunnan | 15 | 98.60 | 27.73 | 1760 |
| NWL | Niwaluo, Gongshan, Yunnan | 21 | 98.57 | 28.03 | 1915 |
| AY | Angying, Jianhe, Guizhou | 10 | 108.34 | 26.37 | 1070 |
| GT | Getou, Leishan, Guizhou | 14 | 108.25 | 26.41 | 1043 |
| GU | Gutian, Fujian | 10 | 119.18 | 26.60 | 757 |
| YX | Youxi, Fujian | 5 | 118.43 | 26.04 | 860 |
| PN | Pingnan, Fujian | 9 | 118.81 | 26.94 | 895 |
| LC | Lichuan, Hubei | 15 | 109.08 | 30.08 | 868 |
| TW | Xinzhu, Taiwan | 18 | 121.10 | 24.57 | 902 |
| Total |  | 122 |  |  |  |

**Table S2** The 47 occurrence records of *Taiwania cryptomerioides* used for ecological niche modeling.

| Number | Location | Latitude (°N) | Longitude (°E) |
| --- | --- | --- | --- |
| 1 | Youyang, Chongqing | 29.10 | 108.91 |
| 2 | Xiayang, Gutian, Fujian | 26.54 | 118.66 |
| 3 | Nanling, Gutian, Fujian | 26.79 | 118.90 |
| 4 | Louxia, Gutian, Fujian | 26.60 | 119.18 |
| 5 | Futang, Pingnan, Fujian | 26.94 | 118.81 |
| 6 | Huanglin, Youxi, Fujian | 26.07 | 118.38 |
| 7 | Pengxi, Youxi, Fujian | 26.04 | 118.43 |
| 8 | Angying, Jianhe, Guizhou | 26.37 | 108.34 |
| 9 | Getou, Leishan, Guizhou | 26.41 | 108.25 |
| 10 | Lichuan, Hubei | 30.08 | 109.08 |
| 11 | Alishan, Jiayi, Taiwan | 23.53 | 120.77 |
| 12 | Batongguan, Hualian, Taiwan | 23.33 | 121.29 |
| 13 | Jianshan, Hualian, Taiwan | 24.30 | 121.41 |
| 14 | Liwuxi, Hualian, Taiwan | 24.11 | 121.54 |
| 15 | Tailuge1, Hualian, Taiwan | 24.15 | 121.61 |
| 16 | Tailuge2, Hualian, Taiwan | 24.18 | 121.49 |
| 17 | Fenghuangshan, Nantou, Taiwan | 23.69 | 120.80 |
| 18 | Wusongkeng, Nantou, Taiwan | 23.60 | 120.81 |
| 19 | Lianhuachi, Nantou, Taiwan | 23.90 | 120.88 |
| 20 | Wangxiang, Nantou, Taiwan | 23.61 | 120.93 |
| 21 | Dapushan, Taidong, Taiwan | 22.78 | 120.90 |
| 22 | Guanshan, Taidong, Taiwan | 23.21 | 121.08 |
| 23 | Daxueshan, Taizhong, Taiwan | 24.33 | 121.11 |
| 24 | Chuanxingshan, Taizhong, Taiwan | 24.26 | 120.98 |
| 25 | Dajian, Taizhong, Taiwan | 24.23 | 121.31 |
| 26 | Xinzhu, Taiwan | 24.57 | 121.10 |
| 27 | Taipingshan1, Yilan, Taiwan | 24.54 | 121.49 |
| 28 | Taipingshan2, Yilan, Taiwan | 24.49 | 121.53 |
| 29 | Liushuwa, Changning, Yunnan | 24.84 | 99.78 |
| 30 | Fengshan, Fengqing, Yunnan | 24.59 | 99.91 |
| 31 | Zhiziluo, Fugong, Yunnan | 26.55 | 98.92 |
| 32 | Niwaluo, Gongshan, Yunnan | 28.03 | 98.57 |
| 33 | Qiqi, Gongshan, Yunnan | 27.73 | 98.60 |
| 34 | Shanghedong, Lianghe, Yunnan | 24.84 | 98.43 |
| 35 | Damengzhun, Lincang, Yunnan | 23.73 | 100.03 |
| 36 | Daxiaohe, Longling, Yunnan | 24.62 | 98.85 |
| 37 | Jiangjiatang, Longling, Yunnan | 24.59 | 98.68 |
| 38 | Xiangda, Longling, Yunnan | 24.35 | 98.75 |
| 39 | Guanyinsi, Tengchong, Yunnan | 25.08 | 98.50 |
| 40 | Hejiapo, Tengchong, Yunnan | 25.24 | 98.66 |
| 41 | Kuige, Tengchong, Yunnan | 25.01 | 98.44 |
| 42 | Linjiapu, Tengchong, Yunnan | 25.29 | 98.70 |
| 43 | Tiantaishan, Tengchong, Yunnan | 25.40 | 98.72 |
| 44 | Yanjiachong, Tengchong, Yunnan | 25.08 | 98.43 |
| 45 | Xiongbaluo, Weixi, Yunnan | 27.27 | 99.12 |
| 46 | Zhongshanzhai, Yingjiang, Yunnan | 24.93 | 97.92 |
| 47 | Xiamiaodan, Yunlong, Yunnan | 25.64 | 99.13 |

**Table S3** The 19 environmental variables used in this study.

| Environmental variable | | Description | Unit |
| --- | --- | --- | --- |
| Temperature-related | Bio01 | Annual mean temperature | ℃ |
|  | Bio02 | Mean diurnal range | ℃ |
|  | Bio03 | Isothermality (Bio2/Bio7 × 100) | - |
|  | Bio04 | Temperature seasonality | - |
|  | Bio05 | Max temperature of warmest month | ℃ |
|  | Bio06 | Min temperature of coldest month | ℃ |
|  | Bio07 | Temperature annual range | ℃ |
|  | Bio08 | Mean temperature of wettest quarter | ℃ |
|  | Bio09 | Mean temperature of driest quarter | ℃ |
|  | Bio10 | Mean temperature of warmest quarter | ℃ |
|  | Bio11 | Mean temperature of coldest quarter | ℃ |
| Precipitation-related | Bio12 | Annual precipitation | mm |
|  | Bio13 | Precipitation of wettest month | mm |
|  | Bio14 | Precipitation of driest month | mm |
|  | Bio15 | Precipitation seasonality | - |
|  | Bio16 | Precipitation of wettest quarter | mm |
|  | Bio17 | Precipitation of driest quarter | mm |
|  | Bio18 | Precipitation of warmest quarter | mm |
|  | Bio19 | Precipitation of coldest quarter | mm |

**Table S4** Statistics for RAD-seq data of each sampled individual of *Taiwania cryptomerioides*.

| Samples | Raw reads | Raw_base (G) | Clean reads | Clean_base (G) | Depths of coverage |
| --- | --- | --- | --- | --- | --- |
| CQ1 | 25773286 | 3.866 | 25483205 | 3.822 | 8.27 |
| CQ2 | 29059988 | 4.359 | 28470027 | 4.271 | 8.39 |
| CQ3 | 21691004 | 3.254 | 21387739 | 3.208 | 7.98 |
| CQ4 | 21300422 | 3.195 | 20955669 | 3.143 | 7.77 |
| CQ5 | 14848700 | 2.227 | 14672812 | 2.201 | 7.62 |
| QQ1 | 13306188 | 1.996 | 13154549 | 1.973 | 8.07 |
| QQ3 | 18420764 | 2.763 | 18244016 | 2.737 | 8.46 |
| QQ4 | 22123952 | 3.319 | 21933180 | 3.290 | 7.55 |
| QQ6 | 14699848 | 2.205 | 14504785 | 2.176 | 8.46 |
| QQ7 | 9915116 | 1.487 | 9812877 | 1.472 | 11.17 |
| QQ8 | 13457522 | 2.019 | 13264505 | 1.990 | 10.27 |
| QQ9 | 32700824 | 4.905 | 32379973 | 4.857 | 8.46 |
| QQ11 | 29460834 | 4.419 | 29079456 | 4.362 | 7.85 |
| QQ13 | 28099898 | 4.215 | 27758285 | 4.164 | 7.87 |
| QQ15 | 20362368 | 3.054 | 20123382 | 3.019 | 8.17 |
| QQ17 | 20880402 | 3.132 | 20607601 | 3.091 | 7.93 |
| QQ19 | 26289532 | 3.943 | 25943939 | 3.892 | 7.85 |
| QQ21 | 19753268 | 2.963 | 19557248 | 2.934 | 8.32 |
| QQ23 | 19199952 | 2.880 | 18853148 | 2.828 | 8.00 |
| QQ24 | 18320526 | 2.748 | 18149270 | 2.722 | 7.98 |
| NWL1 | 15062550 | 2.259 | 14911596 | 2.237 | 7.56 |
| NWL3 | 16977068 | 2.547 | 16819499 | 2.523 | 7.48 |
| NWL4 | 15665434 | 2.350 | 15524066 | 2.329 | 7.46 |
| NWL6 | 24642126 | 3.696 | 24415408 | 3.662 | 8.44 |
| NWL7 | 21306628 | 3.196 | 21170639 | 3.176 | 8.04 |
| NWL10 | 20023728 | 3.004 | 19899807 | 2.985 | 7.92 |
| NWL12 | 14342456 | 2.151 | 14244397 | 2.137 | 7.46 |
| NWL13 | 10950092 | 1.643 | 10838882 | 1.626 | 7.37 |
| NWL14 | 8181726 | 1.227 | 8126786 | 1.219 | 6.88 |
| NWL16 | 8528010 | 1.279 | 8455409 | 1.268 | 8.05 |
| NWL18 | 19479382 | 2.922 | 19328526 | 2.899 | 7.89 |
| NWL20 | 29037016 | 4.356 | 28656719 | 4.299 | 8.16 |
| NWL22 | 22469366 | 3.370 | 22183967 | 3.328 | 8.06 |
| NWL24 | 15658030 | 2.349 | 15443681 | 2.317 | 7.86 |
| NWL26 | 20443458 | 3.067 | 20231897 | 3.035 | 7.94 |
| NWL15 | 11182068 | 1.677 | 7169795 | 1.075 | 16.28 |
| NWL17 | 8232614 | 1.235 | 7981738 | 1.197 | 14.74 |
| NWL19 | 3723188 | 0.558 | 3497677 | 0.525 | 14.49 |
| NWL21 | 12690654 | 1.904 | 12072729 | 1.811 | 11.27 |
| NWL23 | 5803994 | 0.871 | 5621756 | 0.843 | 11.90 |
| NWL25 | 59217046 | 8.883 | 28688092 | 4.303 | 13.24 |
| AY2 | 6314946 | 0.947 | 5364544 | 0.805 | 6.88 |
| AY4 | 6581296 | 0.987 | 4506014 | 0.676 | 6.23 |
| AY6 | 5522156 | 0.828 | 5404152 | 0.811 | 7.64 |
| AY7 | 3233254 | 0.485 | 3027293 | 0.454 | 12.79 |
| AY9 | 9317888 | 1.398 | 9045345 | 1.357 | 7.29 |
| AY10 | 4619350 | 0.693 | 4480683 | 0.672 | 9.45 |
| AY13 | 30315538 | 4.547 | 23006318 | 3.451 | 7.86 |
| AY14 | 13633062 | 2.045 | 11677614 | 1.752 | 6.87 |
| AY15 | 19639490 | 2.946 | 15320760 | 2.298 | 7.62 |
| AY16 | 14092328 | 2.114 | 13486339 | 2.023 | 7.16 |
| GT2 | 20514906 | 3.077 | 19248860 | 2.887 | 8.64 |
| GT3 | 19952876 | 2.993 | 18776031 | 2.816 | 9.49 |
| GT5 | 27805952 | 4.171 | 26272281 | 3.941 | 8.41 |
| GT7 | 21419812 | 3.213 | 19450608 | 2.918 | 9.45 |
| GT9 | 16947424 | 2.542 | 16116554 | 2.417 | 9.94 |
| GT11 | 24388024 | 3.658 | 23340833 | 3.501 | 7.27 |
| GT12 | 26312376 | 3.947 | 25551449 | 3.833 | 7.49 |
| GT13 | 29069772 | 4.360 | 28119624 | 4.218 | 8.19 |
| GT15 | 20313734 | 3.047 | 19677668 | 2.952 | 7.51 |
| GT17 | 15314966 | 2.297 | 14428549 | 2.164 | 9.52 |
| GT19 | 17267736 | 2.590 | 16085788 | 2.413 | 10.61 |
| GT21 | 20131498 | 3.020 | 19065676 | 2.860 | 8.22 |
| GT22 | 20317456 | 3.048 | 18999977 | 2.850 | 8.28 |
| GT26 | 22023830 | 3.304 | 20907946 | 3.136 | 8.09 |
| GU1 | 19522290 | 2.928 | 18411380 | 2.762 | 8.05 |
| GU2 | 25936372 | 3.890 | 25046824 | 3.757 | 7.49 |
| GU3 | 21119474 | 3.168 | 19754975 | 2.963 | 8.38 |
| GU4 | 19877948 | 2.982 | 18917510 | 2.838 | 8.06 |
| GU5 | 19397228 | 2.910 | 18282034 | 2.742 | 9.10 |
| GU6 | 14814722 | 2.222 | 13377836 | 2.007 | 10.74 |
| GU14 | 17544916 | 2.632 | 15661980 | 2.349 | 11.41 |
| GU15 | 17668618 | 2.650 | 16701254 | 2.505 | 10.23 |
| GU16 | 15802448 | 2.370 | 14886828 | 2.233 | 10.70 |
| GU19 | 17014140 | 2.552 | 16798266 | 2.520 | 7.95 |
| YX1 | 2902710 | 0.435 | 2826284 | 0.424 | 32.36 |
| YX2 | 6736140 | 1.010 | 6555662 | 0.983 | 13.96 |
| YX3 | 6514538 | 0.977 | 6300378 | 0.945 | 10.50 |
| YX4 | 12606362 | 1.891 | 12406799 | 1.861 | 11.56 |
| YX5 | 20821370 | 3.123 | 17008825 | 2.551 | 12.50 |
| PN7 | 32799336 | 4.920 | 32543966 | 4.882 | 11.10 |
| PN8 | 26624644 | 3.994 | 26378736 | 3.957 | 10.46 |
| PN9 | 34646896 | 5.197 | 34361750 | 5.154 | 10.94 |
| PN10 | 29804066 | 4.471 | 29560100 | 4.434 | 10.83 |
| PN11 | 17206158 | 2.581 | 17033854 | 2.555 | 9.41 |
| PN12 | 20058012 | 3.009 | 19901364 | 2.985 | 9.73 |
| PN13 | 22040470 | 3.306 | 21851547 | 3.278 | 9.87 |
| PN17 | 38633200 | 5.795 | 38323774 | 5.749 | 11.18 |
| PN18 | 34186966 | 5.128 | 33699067 | 5.055 | 10.97 |
| LC1 | 32699170 | 4.905 | 32319783 | 4.848 | 10.49 |
| LC2 | 24713674 | 3.707 | 24379003 | 3.657 | 10.98 |
| LC3 | 19823396 | 2.974 | 19522166 | 2.928 | 9.19 |
| LC4 | 39291964 | 5.894 | 38873523 | 5.831 | 11.88 |
| LC5 | 28872584 | 4.331 | 28611537 | 4.292 | 9.77 |
| LC6 | 31189374 | 4.678 | 30822247 | 4.623 | 10.36 |
| LC7 | 28444082 | 4.267 | 28188202 | 4.228 | 9.46 |
| LC8 | 14648194 | 2.197 | 14512674 | 2.177 | 7.07 |
| LC9 | 29059298 | 4.359 | 27447955 | 4.117 | 12.90 |
| LC10 | 19783510 | 2.968 | 18630469 | 2.795 | 9.40 |
| LC11 | 21541316 | 3.231 | 20193533 | 3.029 | 10.29 |
| LC12 | 17700878 | 2.655 | 16334478 | 2.450 | 11.09 |
| LC13 | 17981626 | 2.697 | 16683247 | 2.502 | 10.48 |
| LC14 | 17700612 | 2.655 | 16761600 | 2.514 | 8.95 |
| LC15 | 16005828 | 2.401 | 15470037 | 2.321 | 8.05 |
| TW1-2 | 20446432 | 3.067 | 20146275 | 3.022 | 8.17 |
| TW1-3 | 16647010 | 2.497 | 16464720 | 2.470 | 7.52 |
| TW2-1 | 46706250 | 7.006 | 46273467 | 6.941 | 10.62 |
| TW2-2 | 10356642 | 1.553 | 10223970 | 1.534 | 6.59 |
| TW2-3 | 9827348 | 1.474 | 9667928 | 1.450 | 6.73 |
| TW2-4 | 23784038 | 3.568 | 23549851 | 3.532 | 8.61 |
| TW2-5 | 7276174 | 1.091 | 7077257 | 1.062 | 6.32 |
| TW2-6 | 34831190 | 5.225 | 34428026 | 5.164 | 9.68 |
| TW7 | 3834850 | 0.575 | 3639947 | 0.546 | 15.41 |
| TW8 | 8474058 | 1.271 | 8071623 | 1.211 | 9.32 |
| TW10 | 13839488 | 2.076 | 13388685 | 2.008 | 11.25 |
| TW11 | 17612244 | 2.642 | 17072486 | 2.561 | 11.30 |
| TW12 | 16864614 | 2.530 | 16048856 | 2.407 | 11.45 |
| TW13 | 16602734 | 2.490 | 15881370 | 2.382 | 12.44 |
| TW14 | 3547838 | 0.532 | 3424603 | 0.514 | 14.19 |
| TW16 | 1931796 | 0.290 | 1872735 | 0.281 | 19.28 |
| TW17 | 558410 | 0.084 | 525976 | 0.079 | 31.34 |
| TW18 | 2257514 | 0.339 | 2196417 | 0.329 | 20.86 |
| Mean | 18913638 | 2.837 | 18002256 | 2.700 | 9.88 |

**Table S5** Genetic differentiation (*F*_ST_) between three genetic groups of *Taiwania cryptomerioides.*

| Group | Southwest | Central-eastern | Taiwan |
| --- | --- | --- | --- |
| Southwest | – | 0.20 | 0.33 |
| Central-eastern | 0.20 | – | 0.22 |
| Taiwan | 0.33 | 0.22 | – |

**Table S6** Relative likelihood of the four demographic models tested in this study.

| Model | log_10_(Likelihood) | AIC_i_ | Δ_i_ | w_i_ |
| --- | --- | --- | --- | --- |
| Model1 | -381.47 | 1770.74 | 76.346 | 0 |
| Model2 | -388.70 | 1806.02 | 83.574 | 0 |
| Model3 | -363.52 | 1690.08 | 58.396 | 1 |
| Model4 | -386.44 | 1795.65 | 81.321 | 0 |

*Note:* log_10_(Likelihood) is the highest value among the 50 independent runs for each model. AIC_i_, Δ_i_ and w_i_ were calculated following the methods shown in Excoffier et al. (2013).

**Table S7** The potential distribution areas of *Taiwania cryptomerioides* in the different periods and climate scenarios.

| Period | Low suitability | | Medium suitability | | High suitability | | Total suitable |
| --- | --- | --- | --- | --- | --- | --- | --- |
|  | Area (10^4^ km^2^) | Percentage (%) | Area (10^4^ km^2^) | Percentage (%) | Area (10^4^ km^2^) | Percentage (%) | Area (10^4^ km^2^) |
| Current | 9.46 | 58.94 | 3.39 | 21.12 | 3.20 | 19.94 | 16.05 |
| 2030s_SSP245 | 6.45 | 60.96 | 2.09 | 19.75 | 2.04 | 19.28 | 10.58 |
| 2030s_SSP585 | 4.03 | 50.00 | 2.02 | 25.06 | 2.01 | 24.94 | 8.06 |
| 2070s_SSP245 | 7.44 | 62.26 | 2.38 | 19.92 | 2.13 | 17.82 | 11.95 |
| 2070s_SSP585 | 2.97 | 45.14 | 1.67 | 25.38 | 1.94 | 29.48 | 6.58 |

**Table S8** The outlier loci identified with BayeScan.

| Locus | prob | log10.PO. | qval | alpha | fst |
| --- | --- | --- | --- | --- | --- |
| SNP4 | 0.9991 | 3.0473 | 0.00043807 | 2.0293 | 0.83001 |
| SNP6 | 0.93937 | 1.1901 | 0.020391 | -1.6606 | 0.17557 |
| SNP32 | 0.84349 | 0.73153 | 0.048959 | -1.3291 | 0.2233 |
| SNP46 | 0.99552 | 2.3467 | 0.0013039 | -1.8152 | 0.15116 |
| SNP58 | 0.96207 | 1.4042 | 0.010594 | -1.6688 | 0.17174 |
| SNP79 | 0.97581 | 1.6057 | 0.0074174 | -1.9302 | 0.14376 |
| SNP100 | 0.99851 | 2.8252 | 0.00060544 | -2.03 | 0.13027 |
| SNP110 | 0.98865 | 1.94 | 0.0027848 | -1.7829 | 0.1563 |
| SNP112 | 0.9997 | 3.5247 | 0.0001757 | 2.0417 | 0.83154 |
| SNP122 | 0.98118 | 1.7172 | 0.0058471 | -1.6988 | 0.16605 |
| SNP139 | 0.99134 | 2.0586 | 0.002086 | 1.5991 | 0.77157 |
| SNP147 | 0.99432 | 2.2436 | 0.0017871 | -1.9269 | 0.14097 |
| SNP148 | 0.8644 | 0.80444 | 0.043883 | -1.1567 | 0.24259 |
| SNP167 | 0.89188 | 0.91638 | 0.037744 | -1.1862 | 0.23636 |
| SNP173 | 0.98746 | 1.8961 | 0.003556 | -1.5815 | 0.17826 |
| SNP179 | 0.85066 | 0.75557 | 0.046788 | -1.1149 | 0.24883 |
| SNP180 | 0.99761 | 2.6207 | 0.00077516 | -1.8037 | 0.15113 |
| SNP201 | 0.85544 | 0.77213 | 0.04553 | -1.5796 | 0.19534 |
| SNP214 | 0.88262 | 0.87616 | 0.039423 | -1.331 | 0.21912 |
| SNP262 | 0.9782 | 1.6519 | 0.0067509 | 1.5223 | 0.75868 |
| SNP274 | 0.99253 | 2.1236 | 0.0018817 | 1.6347 | 0.77745 |
| SNP276 | 0.93309 | 1.1445 | 0.025235 | -1.1999 | 0.23185 |
| SNP287 | 0.98596 | 1.8465 | 0.0042899 | -1.7905 | 0.15567 |
| SNP290 | 0.9991 | 3.0473 | 0.00043807 | 1.988 | 0.82332 |
| SNP295 | 0.9991 | 3.0473 | 0.00043807 | 1.7258 | 0.79118 |
| SNP336 | 0.85693 | 0.7774 | 0.045118 | 1.1675 | 0.69638 |
| SNP338 | 0.99432 | 2.2436 | 0.0017871 | 1.7179 | 0.78907 |
| SNP342 | 0.9991 | 3.0473 | 0.00043807 | 2.0362 | 0.83059 |
| SNP360 | 0.94086 | 1.2016 | 0.019035 | -1.4034 | 0.20458 |
| SNP377 | 0.86708 | 0.81449 | 0.042724 | -1.2543 | 0.22908 |
| SNP381 | 0.94176 | 1.2087 | 0.017921 | 1.5869 | 0.76307 |
| SNP391 | 0.84498 | 0.73647 | 0.048526 | -1.2407 | 0.23337 |
| SNP405 | 0.98327 | 1.7693 | 0.0054368 | -1.446 | 0.19529 |
| SNP412 | 0.99671 | 2.482 | 0.00096099 | -1.8067 | 0.1523 |
| SNP421 | 0.9997 | 3.5247 | 0.0001757 | 1.9416 | 0.8186 |
| SNP426 | 0.9994 | 3.2235 | 0.00027153 | 1.9912 | 0.82442 |
| SNP447 | 0.9994 | 3.2235 | 0.00027153 | 2.033 | 0.83042 |
| SNP448 | 0.94803 | 1.2611 | 0.014703 | 1.3354 | 0.72894 |
| SNP455 | 0.91876 | 1.0534 | 0.032215 | 1.2374 | 0.71077 |
| SNP457 | 0.94205 | 1.2111 | 0.017057 | 1.5526 | 0.75853 |
| SNP460 | 0.8853 | 0.88755 | 0.03873 | -1.4844 | 0.20168 |
| SNP479 | 1 | 1000 | 0 | -2.1008 | 0.12026 |
| SNP496 | 0.9991 | 3.0473 | 0.00043807 | 1.9703 | 0.82211 |
| SNP512 | 0.93041 | 1.1261 | 0.027172 | 1.5238 | 0.75287 |
| SNP520 | 0.95579 | 1.3349 | 0.012156 | -1.7248 | 0.16683 |
| SNP530 | 0.93757 | 1.1766 | 0.021959 | 1.3182 | 0.72522 |
| SNP583 | 0.99881 | 2.9222 | 0.00052709 | -1.8163 | 0.14933 |
| SNP604 | 0.89098 | 0.91236 | 0.038067 | -1.2331 | 0.23053 |
| SNP605 | 0.9328 | 1.1424 | 0.02572 | 1.1227 | 0.69264 |
| SNP633 | 0.9997 | 3.5247 | 0.0001757 | -2.1048 | 0.12199 |
| SNP637 | 0.9083 | 0.99588 | 0.035084 | -1.2049 | 0.23262 |
| SNP641 | 0.99612 | 2.4092 | 0.0012416 | 1.6748 | 0.78284 |
| SNP645 | 0.9328 | 1.1424 | 0.02572 | 1.511 | 0.7517 |
| SNP647 | 0.92772 | 1.1084 | 0.028834 | -1.625 | 0.18036 |
| SNP651 | 0.96834 | 1.4855 | 0.0087222 | 1.2089 | 0.70941 |
| SNP652 | 0.93668 | 1.17 | 0.02273 | 1.5572 | 0.75817 |
| SNP712 | 0.95818 | 1.3601 | 0.011363 | -1.5276 | 0.18805 |
| SNP726 | 0.9997 | 3.5247 | 0.0001757 | 2.0279 | 0.83006 |
| SNP734 | 0.92503 | 1.0913 | 0.030012 | 1.4746 | 0.74609 |
| SNP772 | 0.99462 | 2.2672 | 0.0015788 | 1.6703 | 0.78202 |
| SNP809 | 0.99821 | 2.7459 | 0.0006663 | 1.7239 | 0.7909 |
| SNP833 | 0.97103 | 1.5252 | 0.0082934 | -1.4035 | 0.20173 |
| SNP842 | 0.95102 | 1.2881 | 0.013264 | -1.2439 | 0.22437 |
| SNP845 | 0.95131 | 1.2909 | 0.012981 | 1.3294 | 0.72781 |
| SNP897 | 0.93757 | 1.1766 | 0.021959 | -1.2305 | 0.2274 |
| SNP917 | 0.99104 | 2.0438 | 0.0023008 | -1.7815 | 0.1553 |
| SNP938 | 0.85514 | 0.77108 | 0.045941 | -1.4969 | 0.20301 |
| SNP987 | 0.86619 | 0.81112 | 0.043494 | -1.2592 | 0.22924 |
| SNP1012 | 0.98357 | 1.7772 | 0.0053085 | -1.9242 | 0.14411 |
| SNP1017 | 0.84259 | 0.72859 | 0.049393 | -1.2148 | 0.23654 |
| SNP1023 | 0.94415 | 1.228 | 0.015583 | 1.5529 | 0.75808 |
| SNP1051 | 0.97969 | 1.6834 | 0.0062913 | -1.7467 | 0.16182 |
| SNP1061 | 0.98088 | 1.7102 | 0.0059898 | 1.4729 | 0.75235 |
| SNP1098 | 0.89247 | 0.91908 | 0.037423 | -1.7169 | 0.17751 |
| SNP1100 | 0.92354 | 1.082 | 0.030491 | 1.5214 | 0.75215 |
| SNP1148 | 0.93877 | 1.1856 | 0.021182 | 1.542 | 0.75673 |
| SNP1150 | 0.99881 | 2.9222 | 0.00052709 | -1.931 | 0.13839 |
| SNP1163 | 0.87007 | 0.82585 | 0.041958 | -1.0177 | 0.26177 |
| SNP1173 | 0.98566 | 1.8373 | 0.0044139 | 1.7921 | 0.79593 |
| SNP1177 | 0.99731 | 2.5694 | 0.00086212 | 1.7266 | 0.79093 |
| SNP1181 | 0.93668 | 1.17 | 0.02273 | 1.2795 | 0.71844 |
| SNP1210 | 0.99612 | 2.4092 | 0.0012416 | -1.8345 | 0.14881 |
| SNP1269 | 0.93937 | 1.1901 | 0.020391 | -1.2792 | 0.22038 |
| SNP1278 | 0.9997 | 3.5247 | 0.0001757 | 1.9512 | 0.81991 |
| SNP1294 | 0.98536 | 1.8282 | 0.0046602 | 1.59 | 0.7694 |
| SNP1299 | 0.9098 | 1.0037 | 0.034814 | -1.1492 | 0.24051 |
| SNP1320 | 0.85066 | 0.75557 | 0.046788 | 1.1248 | 0.68918 |
| SNP1321 | 0.99821 | 2.7459 | 0.0006663 | -2.0747 | 0.12447 |
| SNP1341 | 0.90502 | 0.97902 | 0.035912 | 1.1265 | 0.69206 |
| SNP1362 | 0.9307 | 1.1281 | 0.026693 | 1.5468 | 0.75637 |
| SNP1365 | 0.91249 | 1.0181 | 0.033491 | 1.2482 | 0.71261 |
| SNP1386 | 0.98955 | 1.9762 | 0.002657 | 1.6039 | 0.77218 |
| SNP1393 | 0.90621 | 0.98509 | 0.035635 | 1.2429 | 0.71137 |
| SNP1397 | 0.99462 | 2.2672 | 0.0015788 | -1.6795 | 0.16647 |
| SNP1415 | 0.91667 | 1.0414 | 0.032961 | 1.4935 | 0.74757 |
| SNP1419 | 0.89695 | 0.93974 | 0.036793 | -1.2108 | 0.23269 |
| SNP1426 | 0.93668 | 1.17 | 0.02273 | 1.5372 | 0.75465 |
| SNP1438 | 0.93429 | 1.1528 | 0.023992 | 1.4223 | 0.73987 |
| SNP1439 | 0.88053 | 0.86747 | 0.04012 | -1.2351 | 0.23093 |
| SNP1443 | 0.99851 | 2.8252 | 0.00060544 | -1.9727 | 0.13532 |
| SNP1462 | 0.95579 | 1.3349 | 0.012156 | -1.4762 | 0.19439 |
| SNP1466 | 0.98178 | 1.7315 | 0.0057046 | 1.5796 | 0.76739 |
| SNP1472 | 0.91786 | 1.0482 | 0.032463 | 1.2915 | 0.71962 |
| SNP1494 | 0.9319 | 1.1362 | 0.025963 | -1.3694 | 0.20992 |
| SNP1499 | 0.98447 | 1.802 | 0.004916 | -1.6942 | 0.16703 |
| SNP1501 | 0.9997 | 3.5247 | 0.0001757 | 1.9398 | 0.81876 |
| SNP1516 | 0.94982 | 1.2771 | 0.013553 | 1.6012 | 0.76622 |
| SNP1532 | 0.85723 | 0.77845 | 0.044708 | -1.4768 | 0.2047 |
| SNP1548 | 0.91726 | 1.0448 | 0.032712 | -1.4032 | 0.20755 |
| SNP1557 | 0.92413 | 1.0857 | 0.030251 | 1.2411 | 0.71187 |
| SNP1565 | 0.97879 | 1.6642 | 0.0065957 | 1.4403 | 0.74729 |
| SNP1587 | 0.94385 | 1.2255 | 0.015881 | -1.3083 | 0.21645 |
| SNP1605 | 0.92085 | 1.0657 | 0.031223 | 1.5433 | 0.7557 |
| SNP1613 | 0.92264 | 1.0765 | 0.030732 | 1.5226 | 0.75207 |
| SNP1643 | 0.94833 | 1.2637 | 0.014419 | 1.2845 | 0.7201 |
| SNP1650 | 0.94056 | 1.1993 | 0.019308 | -1.2572 | 0.22273 |
| SNP1652 | 0.8417 | 0.72567 | 0.049827 | 1.1255 | 0.68804 |
| SNP1653 | 0.96714 | 1.4689 | 0.0089416 | -1.4151 | 0.20023 |
| SNP1659 | 0.97521 | 1.5948 | 0.007586 | -1.4755 | 0.19269 |
| SNP1685 | 0.92802 | 1.1103 | 0.028362 | 1.5437 | 0.75538 |
| SNP1699 | 0.93967 | 1.1924 | 0.019855 | 1.3218 | 0.72556 |
| SNP1714 | 0.98775 | 1.9067 | 0.0031762 | 1.2333 | 0.71519 |
| SNP1720 | 0.9997 | 3.5247 | 0.0001757 | 2.0363 | 0.83114 |
| SNP1722 | 0.98716 | 1.8857 | 0.0036798 | 1.6146 | 0.77293 |
| SNP1725 | 0.97581 | 1.6057 | 0.0074174 | -1.8961 | 0.14686 |
| SNP1738 | 0.99612 | 2.4092 | 0.0012416 | 1.6963 | 0.78632 |
| SNP1743 | 0.92921 | 1.1182 | 0.027884 | 1.5333 | 0.75418 |
| SNP1746 | 0.98357 | 1.7772 | 0.0053085 | -1.9253 | 0.14263 |
| SNP1755 | 0.94086 | 1.2016 | 0.019035 | 1.4488 | 0.74471 |
| SNP1756 | 0.99432 | 2.2436 | 0.0017871 | 1.6331 | 0.77674 |
| SNP1768 | 0.96326 | 1.4186 | 0.010124 | 1.3978 | 0.73969 |
| SNP1791 | 0.89606 | 0.93554 | 0.037101 | -1.4047 | 0.20944 |
| SNP1792 | 0.98626 | 1.856 | 0.0041665 | -1.5801 | 0.17848 |
| SNP1795 | 0.92772 | 1.1084 | 0.028834 | -1.3407 | 0.21376 |
| SNP1803 | 0.99492 | 2.2921 | 0.0014381 | -1.8913 | 0.14435 |
| SNP1815 | 0.91189 | 1.0149 | 0.033756 | 1.1694 | 0.69985 |
| SNP1817 | 0.92742 | 1.1065 | 0.0293 | -1.2365 | 0.2271 |
| SNP1831 | 0.93339 | 1.1465 | 0.024989 | 1.3013 | 0.72203 |
| SNP1846 | 0.92742 | 1.1065 | 0.0293 | 1.514 | 0.75112 |
| SNP1867 | 0.97969 | 1.6834 | 0.0062913 | 1.3978 | 0.74068 |
| SNP1873 | 0.99522 | 2.3186 | 0.0013694 | -1.7527 | 0.15827 |
| SNP1877 | 0.98357 | 1.7772 | 0.0053085 | 1.5208 | 0.75865 |
| SNP1889 | 0.93967 | 1.1924 | 0.019855 | 1.3178 | 0.72543 |
| SNP1894 | 0.97909 | 1.6705 | 0.0064435 | -1.8221 | 0.1544 |
| SNP1896 | 0.94325 | 1.2207 | 0.016471 | 1.5523 | 0.75855 |
| SNP1903 | 0.96266 | 1.4114 | 0.010359 | -1.7427 | 0.16397 |
| SNP1908 | 0.9994 | 3.2235 | 0.00027153 | 1.9842 | 0.82462 |
| SNP1924 | 0.85783 | 0.78058 | 0.044296 | -1.2186 | 0.23532 |
| SNP1948 | 0.93041 | 1.1261 | 0.027172 | 1.5433 | 0.75665 |
| SNP1967 | 0.95669 | 1.3442 | 0.011627 | 1.1923 | 0.7059 |
| SNP1975 | 0.94922 | 1.2717 | 0.013841 | 1.4819 | 0.75027 |
| SNP1977 | 0.9307 | 1.1281 | 0.026693 | -1.6614 | 0.17669 |
| SNP1997 | 0.9767 | 1.6225 | 0.0070818 | -1.4358 | 0.19721 |
| SNP2020 | 0.94176 | 1.2087 | 0.017921 | 1.5611 | 0.75979 |
| SNP2047 | 0.99104 | 2.0438 | 0.0023008 | 1.6004 | 0.77133 |
| SNP2078 | 0.96446 | 1.4335 | 0.0094139 | 1.4406 | 0.74627 |
| SNP2115 | 1 | 1000 | 0 | 2.0394 | 0.8313 |
| SNP2134 | 0.98805 | 1.9175 | 0.0030466 | -1.6518 | 0.17006 |
| SNP2142 | 0.94146 | 1.2063 | 0.018481 | 1.5584 | 0.75916 |
| SNP2149 | 0.9997 | 3.5247 | 0.0001757 | 1.7791 | 0.80008 |
| SNP2163 | 0.93877 | 1.1856 | 0.021182 | -1.3534 | 0.21103 |
| SNP2174 | 0.9101 | 1.0053 | 0.034549 | 1.4937 | 0.74726 |
| SNP2196 | 0.96834 | 1.4855 | 0.0087222 | -1.688 | 0.16931 |
| SNP2205 | 0.86977 | 0.8247 | 0.042336 | 1.1919 | 0.70042 |
| SNP2225 | 0.95908 | 1.3699 | 0.010851 | 1.3644 | 0.73408 |
| SNP2226 | 0.97461 | 1.5842 | 0.0079251 | 1.4322 | 0.7455 |
| SNP2233 | 0.99791 | 2.6788 | 0.00073579 | 1.5259 | 0.76222 |
| SNP2237 | 0.91129 | 1.0117 | 0.034283 | 1.4545 | 0.74226 |
| SNP2241 | 0.90024 | 0.9554 | 0.036487 | 1.1726 | 0.69965 |
| SNP2244 | 0.96476 | 1.4373 | 0.0091785 | 1.4221 | 0.74338 |
| SNP2248 | 0.93369 | 1.1486 | 0.024494 | 1.5318 | 0.75382 |
| SNP2274 | 0.98686 | 1.8756 | 0.0040437 | 1.5796 | 0.76771 |
| SNP2275 | 1 | 1000 | 0 | 2.1763 | 0.84927 |
| SNP2277 | 0.87694 | 0.85286 | 0.04083 | -1.546 | 0.19645 |
| SNP2294 | 0.99701 | 2.5235 | 0.00090933 | -1.8219 | 0.1504 |
| SNP2321 | 0.99881 | 2.9222 | 0.00052709 | -2.1037 | 0.12125 |
| SNP2325 | 0.93011 | 1.1241 | 0.027409 | 1.5171 | 0.75165 |
| SNP2376 | 0.92055 | 1.064 | 0.031468 | 1.5066 | 0.74908 |
| SNP2389 | 0.99791 | 2.6788 | 0.00073579 | 1.741 | 0.79294 |
| SNP2406 | 0.88411 | 0.88246 | 0.039075 | -1.5766 | 0.19156 |
| SNP2429 | 0.9994 | 3.2235 | 0.00027153 | 2.0071 | 0.82692 |
| SNP2431 | 0.93638 | 1.1679 | 0.022982 | 1.5683 | 0.75966 |
| SNP2437 | 0.87007 | 0.82585 | 0.041958 | -1.316 | 0.22157 |
| SNP2457 | 0.94773 | 1.2584 | 0.014986 | 1.319 | 0.72599 |
| SNP2462 | 0.9991 | 3.0473 | 0.00043807 | 2.0166 | 0.82814 |
| SNP2480 | 0.91876 | 1.0534 | 0.032215 | 1.5042 | 0.74914 |
| SNP2553 | 0.98536 | 1.8282 | 0.0046602 | 1.5628 | 0.76533 |
| SNP2560 | 0.9991 | 3.0473 | 0.00043807 | 2.0334 | 0.83014 |
| SNP2561 | 0.92593 | 1.0969 | 0.029537 | 1.5514 | 0.75698 |
| SNP2578 | 1 | 1000 | 0 | 2.0254 | 0.82935 |
| SNP2583 | 0.9316 | 1.1342 | 0.026206 | 1.5407 | 0.75491 |
| SNP2593 | 0.9543 | 1.3198 | 0.012427 | -1.3241 | 0.21309 |
| SNP2595 | 0.9997 | 3.5247 | 0.0001757 | 2.0174 | 0.82837 |
| SNP2599 | 0.99612 | 2.4092 | 0.0012416 | 1.4528 | 0.75112 |
| SNP2607 | 0.93877 | 1.1856 | 0.021182 | 1.5597 | 0.75875 |
| SNP2667 | 0.95878 | 1.3666 | 0.011107 | -1.8335 | 0.15575 |
| SNP2677 | 0.92832 | 1.1123 | 0.028124 | 1.5015 | 0.74997 |
| SNP2678 | 0.93399 | 1.1507 | 0.024244 | 1.55 | 0.75704 |
| SNP2682 | 0.94534 | 1.2379 | 0.015282 | -1.383 | 0.20724 |
| SNP2730 | 0.94176 | 1.2087 | 0.017921 | 1.5724 | 0.76101 |
| SNP2744 | 0.91308 | 1.0214 | 0.033226 | 1.5021 | 0.74933 |
| SNP2756 | 0.98447 | 1.802 | 0.004916 | -1.5867 | 0.17803 |
| SNP2758 | 0.93608 | 1.1657 | 0.023233 | -1.5889 | 0.18332 |
| SNP2760 | 0.9767 | 1.6225 | 0.0070818 | -1.5663 | 0.1817 |
| SNP2761 | 0.93519 | 1.1592 | 0.023487 | 1.5518 | 0.75729 |
| SNP2776 | 0.99881 | 2.9222 | 0.00052709 | 2.0255 | 0.82949 |
| SNP2799 | 0.97372 | 1.5687 | 0.0080983 | 1.445 | 0.74772 |
| SNP2813 | 0.9994 | 3.2235 | 0.00027153 | 1.9899 | 0.82567 |
| SNP2821 | 0.9991 | 3.0473 | 0.00043807 | 1.958 | 0.82086 |
| SNP2831 | 0.9997 | 3.5247 | 0.0001757 | 2.0464 | 0.83194 |
| SNP2891 | 0.93847 | 1.1833 | 0.02144 | 1.5711 | 0.75992 |
| SNP2914 | 0.99223 | 2.1064 | 0.0019782 | 1.553 | 0.76474 |
| SNP2915 | 0.92951 | 1.1201 | 0.027647 | 1.5179 | 0.75216 |
| SNP2931 | 0.98297 | 1.7615 | 0.0055655 | 1.7536 | 0.79048 |
| SNP2935 | 0.94355 | 1.2231 | 0.016177 | 1.5813 | 0.76291 |
| SNP2939 | 0.93489 | 1.1571 | 0.023739 | 1.5827 | 0.76233 |
| SNP2941 | 0.87575 | 0.84807 | 0.041193 | 1.1783 | 0.69892 |
| SNP2959 | 0.84528 | 0.73746 | 0.048094 | 1.1262 | 0.6889 |
| SNP2989 | 0.94146 | 1.2063 | 0.018481 | 1.5734 | 0.76109 |
| SNP3008 | 0.98746 | 1.8961 | 0.003556 | 1.7692 | 0.79255 |
| SNP3014 | 0.98805 | 1.9175 | 0.0030466 | 1.5392 | 0.76221 |
| SNP3021 | 0.98955 | 1.9762 | 0.002657 | 1.5978 | 0.77099 |
| SNP3031 | 0.91159 | 1.0133 | 0.03402 | 1.4087 | 0.73644 |
| SNP3036 | 0.93339 | 1.1465 | 0.024989 | 1.5148 | 0.75199 |
| SNP3040 | 0.96326 | 1.4186 | 0.010124 | -1.5135 | 0.18914 |
| SNP3067 | 0.954 | 1.3168 | 0.012695 | -1.5059 | 0.19079 |
| SNP3112 | 0.86649 | 0.81224 | 0.04311 | -1.6771 | 0.18375 |
| SNP3134 | 0.88232 | 0.87491 | 0.039769 | -1.3397 | 0.21782 |
| SNP3141 | 0.88023 | 0.86624 | 0.040469 | 1.1085 | 0.68775 |
| SNP3145 | 0.99731 | 2.5694 | 0.00086212 | 1.7186 | 0.78942 |
| SNP3157 | 1 | 1000 | 0 | -2.2129 | 0.10924 |
| SNP3169 | 0.99851 | 2.8252 | 0.00060544 | -2.2348 | 0.11206 |
| SNP3173 | 1 | 1000 | 0 | 1.7362 | 0.79533 |
| SNP3214 | 0.84588 | 0.73944 | 0.047661 | -1.4276 | 0.21171 |
| SNP3219 | 0.91876 | 1.0534 | 0.032215 | 1.4772 | 0.74537 |
| SNP3226 | 0.92145 | 1.0693 | 0.030978 | 1.2598 | 0.71498 |
| SNP3227 | 0.94833 | 1.2637 | 0.014419 | 1.4845 | 0.75029 |
| SNP3236 | 0.90382 | 0.97301 | 0.036192 | -1.5841 | 0.18829 |
| SNP3249 | 0.98686 | 1.8756 | 0.0040437 | -1.6702 | 0.16924 |
| SNP3251 | 0.98686 | 1.8756 | 0.0040437 | -1.7546 | 0.15928 |
| SNP3252 | 0.84588 | 0.73944 | 0.047661 | -1.2414 | 0.23316 |
| SNP3299 | 1 | 1000 | 0 | -1.8364 | 0.14674 |
| SNP3302 | 0.92503 | 1.0913 | 0.030012 | -1.4768 | 0.19686 |
| SNP3308 | 0.99014 | 2.002 | 0.0024171 | -1.7221 | 0.16112 |
| SNP3316 | 0.96356 | 1.4223 | 0.0096531 | -1.5939 | 0.1794 |
| SNP3318 | 0.99642 | 2.444 | 0.0010168 | 1.6772 | 0.78353 |
| SNP3325 | 0.97461 | 1.5842 | 0.0079251 | -1.9267 | 0.14454 |
| SNP3327 | 0.89068 | 0.91103 | 0.038388 | -1.6409 | 0.18377 |
| SNP3329 | 0.98746 | 1.8961 | 0.003556 | -1.98 | 0.13718 |
| SNP3338 | 0.94295 | 1.2182 | 0.016763 | -1.6736 | 0.17412 |
| SNP3345 | 0.90621 | 0.98509 | 0.035635 | -1.531 | 0.19389 |

**Table S9** The outlier loci identified with PCAdapt.

| Locus | qval | Locus | qval | Locus | qval |
| --- | --- | --- | --- | --- | --- |
| SNP4 | 3.49E-54 | SNP1314 | 2.20E-12 | SNP2288 | 4.34E-06 |
| SNP29 | 0.006432122 | SNP1319 | 0.008484719 | SNP2292 | 5.27E-05 |
| SNP36 | 2.57E-05 | SNP1320 | 5.00E-05 | SNP2295 | 0.01970835 |
| SNP50 | 0.04464288 | SNP1333 | 4.38E-06 | SNP2299 | 1.38E-05 |
| SNP65 | 4.29E-22 | SNP1336 | 0.012667054 | SNP2306 | 6.61E-13 |
| SNP67 | 0.036300002 | SNP1340 | 1.51E-07 | SNP2325 | 4.64E-21 |
| SNP68 | 0.03015344 | SNP1341 | 1.23E-09 | SNP2332 | 8.78E-12 |
| SNP72 | 2.49E-18 | SNP1348 | 0.004130562 | SNP2336 | 0.013264 |
| SNP104 | 0.000972365 | SNP1353 | 0.013264 | SNP2350 | 4.45E-05 |
| SNP112 | 1.17E-62 | SNP1362 | 3.30E-12 | SNP2354 | 2.16E-06 |
| SNP126 | 1.09E-05 | SNP1364 | 0.00016508 | SNP2358 | 0.003172816 |
| SNP132 | 0.007765318 | SNP1365 | 0.02491048 | SNP2376 | 2.09E-10 |
| SNP138 | 2.19E-05 | SNP1372 | 0.022413294 | SNP2384 | 0.003053485 |
| SNP139 | 0.041509549 | SNP1386 | 5.39E-05 | SNP2389 | 0.005617784 |
| SNP171 | 0.000551692 | SNP1387 | 7.74E-06 | SNP2398 | 0.000983668 |
| SNP174 | 0.014305285 | SNP1389 | 0.013974369 | SNP2429 | 1.42E-30 |
| SNP177 | 0.014009112 | SNP1393 | 4.93E-13 | SNP2431 | 6.40E-10 |
| SNP187 | 1.14E-08 | SNP1406 | 2.22E-05 | SNP2442 | 1.13E-06 |
| SNP191 | 0.015026299 | SNP1409 | 0.014920398 | SNP2450 | 3.38E-07 |
| SNP198 | 0.000596192 | SNP1415 | 1.27E-11 | SNP2452 | 5.98E-07 |
| SNP212 | 0.043440095 | SNP1426 | 1.64E-19 | SNP2457 | 0.023409279 |
| SNP254 | 0.029585559 | SNP1438 | 0.000243657 | SNP2462 | 2.96E-28 |
| SNP262 | 2.37E-08 | SNP1448 | 0.049855234 | SNP2468 | 1.03E-05 |
| SNP274 | 0.020181199 | SNP1450 | 4.65E-05 | SNP2480 | 9.77E-10 |
| SNP290 | 6.52E-30 | SNP1466 | 1.23E-16 | SNP2482 | 1.90E-13 |
| SNP302 | 0.022799493 | SNP1472 | 5.24E-07 | SNP2501 | 0.005491373 |
| SNP313 | 6.16E-06 | SNP1474 | 4.93E-09 | SNP2506 | 0.035054096 |
| SNP318 | 0.000354118 | SNP1477 | 0.02751676 | SNP2510 | 0.001319226 |
| SNP328 | 1.16E-10 | SNP1493 | 4.28E-14 | SNP2515 | 1.33E-11 |
| SNP329 | 0.040104703 | SNP1501 | 2.41E-27 | SNP2540 | 0.049965344 |
| SNP336 | 0.000925909 | SNP1506 | 0.000562893 | SNP2549 | 0.000303497 |
| SNP338 | 2.26E-07 | SNP1516 | 5.75E-21 | SNP2550 | 0.005862777 |
| SNP342 | 2.81E-22 | SNP1525 | 5.36E-05 | SNP2553 | 5.53E-10 |
| SNP345 | 0.01844554 | SNP1529 | 1.95E-08 | SNP2556 | 7.48E-09 |
| SNP353 | 5.12E-06 | SNP1531 | 0.029540493 | SNP2559 | 0.000353361 |
| SNP355 | 0.000698512 | SNP1541 | 0.000627182 | SNP2560 | 4.63E-21 |
| SNP356 | 0.047672259 | SNP1547 | 0.039071123 | SNP2561 | 3.94E-10 |
| SNP359 | 0.035556222 | SNP1564 | 0.008573131 | SNP2572 | 2.09E-18 |
| SNP364 | 0.03958663 | SNP1565 | 1.28E-09 | SNP2578 | 9.21E-69 |
| SNP371 | 0.004142188 | SNP1578 | 0.000611174 | SNP2583 | 6.73E-15 |
| SNP378 | 0.002442375 | SNP1591 | 0.000953074 | SNP2595 | 1.22E-42 |
| SNP380 | 0.010215085 | SNP1603 | 6.98E-05 | SNP2597 | 1.86E-07 |
| SNP381 | 7.86E-31 | SNP1605 | 1.24E-10 | SNP2599 | 0.010713607 |
| SNP403 | 0.011516235 | SNP1613 | 5.50E-13 | SNP2607 | 3.44E-24 |
| SNP420 | 0.039826809 | SNP1615 | 0.015015876 | SNP2610 | 1.03E-05 |
| SNP421 | 1.57E-24 | SNP1624 | 3.36E-05 | SNP2628 | 0.003230306 |
| SNP422 | 0.041069077 | SNP1626 | 0.039071123 | SNP2630 | 0.001841541 |
| SNP424 | 0.018406363 | SNP1631 | 0.000698512 | SNP2643 | 0.003053485 |
| SNP426 | 1.38E-13 | SNP1636 | 3.02E-06 | SNP2654 | 0.031123462 |
| SNP430 | 0.043444415 | SNP1643 | 1.57E-07 | SNP2662 | 0.045524707 |
| SNP435 | 0.00038015 | SNP1652 | 0.004889744 | SNP2665 | 0.027988817 |
| SNP444 | 0.041681526 | SNP1654 | 0.027988817 | SNP2670 | 0.001985722 |
| SNP447 | 1.97E-18 | SNP1655 | 0.034367756 | SNP2677 | 1.12E-07 |
| SNP448 | 0.034367756 | SNP1665 | 0.000530589 | SNP2678 | 2.01E-07 |
| SNP455 | 0.014111736 | SNP1672 | 0.000462082 | SNP2689 | 0.002500327 |
| SNP457 | 6.52E-28 | SNP1685 | 3.68E-09 | SNP2691 | 0.008373838 |
| SNP467 | 2.11E-10 | SNP1686 | 0.003016191 | SNP2698 | 1.22E-10 |
| SNP469 | 4.83E-07 | SNP1699 | 1.07E-10 | SNP2699 | 0.032877202 |
| SNP472 | 0.00010659 | SNP1716 | 2.00E-17 | SNP2720 | 0.043793056 |
| SNP496 | 1.69E-09 | SNP1720 | 1.86E-126 | SNP2723 | 0.005328687 |
| SNP512 | 2.67E-11 | SNP1722 | 6.75E-35 | SNP2730 | 1.71E-11 |
| SNP523 | 0.000878089 | SNP1738 | 2.72E-11 | SNP2744 | 1.01E-07 |
| SNP530 | 3.84E-07 | SNP1740 | 1.39E-08 | SNP2745 | 0.003613174 |
| SNP540 | 0.004384501 | SNP1743 | 5.45E-10 | SNP2750 | 0.000449074 |
| SNP563 | 0.04696286 | SNP1753 | 2.07E-10 | SNP2761 | 8.91E-12 |
| SNP579 | 0.001446074 | SNP1755 | 0.020527816 | SNP2775 | 0.002203316 |
| SNP589 | 6.78E-20 | SNP1756 | 0.011477426 | SNP2776 | 3.60E-45 |
| SNP598 | 3.35E-06 | SNP1763 | 6.20E-06 | SNP2792 | 3.17E-83 |
| SNP609 | 0.035121346 | SNP1768 | 3.10E-07 | SNP2799 | 0.007818405 |
| SNP641 | 1.43E-14 | SNP1783 | 5.12E-07 | SNP2806 | 0.024168327 |
| SNP645 | 6.07E-11 | SNP1789 | 5.81E-19 | SNP2808 | 0.001184151 |
| SNP646 | 0.013225666 | SNP1813 | 1.08E-05 | SNP2813 | 7.37E-16 |
| SNP652 | 1.50E-10 | SNP1815 | 0.00123243 | SNP2814 | 6.05E-05 |
| SNP656 | 0.022634249 | SNP1818 | 0.000986834 | SNP2821 | 5.27E-50 |
| SNP682 | 0.039834166 | SNP1827 | 0.015629498 | SNP2831 | 1.09E-14 |
| SNP694 | 3.94E-07 | SNP1831 | 0.017065959 | SNP2832 | 1.93E-09 |
| SNP699 | 0.020832786 | SNP1846 | 3.44E-08 | SNP2858 | 0.047672259 |
| SNP708 | 0.00016208 | SNP1859 | 7.10E-09 | SNP2867 | 0.000140826 |
| SNP717 | 1.38E-05 | SNP1867 | 0.000199702 | SNP2868 | 4.66E-07 |
| SNP720 | 2.61E-11 | SNP1877 | 1.55E-28 | SNP2882 | 0.033551624 |
| SNP726 | 1.98E-15 | SNP1889 | 0.000671581 | SNP2883 | 4.44E-08 |
| SNP734 | 5.60E-13 | SNP1896 | 2.35E-29 | SNP2888 | 0.000678699 |
| SNP742 | 0.000653853 | SNP1898 | 0.041509549 | SNP2891 | 2.60E-13 |
| SNP747 | 0.041421868 | SNP1899 | 0.000142883 | SNP2901 | 0.000983668 |
| SNP765 | 0.022326834 | SNP1905 | 0.008727099 | SNP2914 | 0.003053485 |
| SNP772 | 4.14E-08 | SNP1908 | 3.94E-09 | SNP2915 | 3.77E-11 |
| SNP784 | 1.00E-07 | SNP1909 | 1.33E-11 | SNP2921 | 8.26E-08 |
| SNP793 | 0.003053485 | SNP1910 | 0.002288334 | SNP2925 | 0.010867656 |
| SNP797 | 0.000500871 | SNP1927 | 0.028020645 | SNP2927 | 5.01E-91 |
| SNP808 | 2.02E-06 | SNP1929 | 0.000105879 | SNP2931 | 0.0010785 |
| SNP809 | 6.18E-07 | SNP1935 | 0.002148403 | SNP2934 | 1.11E-13 |
| SNP845 | 4.77E-05 | SNP1939 | 3.88E-10 | SNP2935 | 5.73E-23 |
| SNP866 | 0.001205491 | SNP1942 | 0.004876468 | SNP2939 | 3.35E-26 |
| SNP867 | 0.042779302 | SNP1946 | 7.88E-08 | SNP2940 | 0.000173761 |
| SNP894 | 0.024168327 | SNP1948 | 5.03E-12 | SNP2941 | 3.05E-16 |
| SNP904 | 0.000499047 | SNP1958 | 3.31E-06 | SNP2950 | 0.004142188 |
| SNP920 | 0.000825667 | SNP1960 | 0.001045621 | SNP2954 | 0.031637688 |
| SNP934 | 0.009872347 | SNP1974 | 0.009022997 | SNP2957 | 4.24E-05 |
| SNP936 | 0.041681526 | SNP1975 | 0.00139747 | SNP2958 | 3.88E-10 |
| SNP945 | 0.016282071 | SNP1996 | 0.039783541 | SNP2959 | 2.18E-06 |
| SNP953 | 1.47E-14 | SNP1998 | 0.008550115 | SNP2975 | 0.007518502 |
| SNP954 | 0.003201627 | SNP2015 | 0.006605063 | SNP2989 | 4.63E-21 |
| SNP957 | 0.004190517 | SNP2020 | 1.33E-11 | SNP3001 | 1.00E-16 |
| SNP961 | 0.010102057 | SNP2029 | 0.022326834 | SNP3014 | 0.007326819 |
| SNP963 | 4.57E-09 | SNP2043 | 0.000738261 | SNP3016 | 0.000182841 |
| SNP984 | 2.71E-09 | SNP2047 | 4.68E-05 | SNP3021 | 9.15E-23 |
| SNP986 | 2.04E-10 | SNP2050 | 5.06E-08 | SNP3022 | 0.039071123 |
| SNP1003 | 0.006117095 | SNP2051 | 0.045482643 | SNP3027 | 0.034367756 |
| SNP1010 | 0.000371973 | SNP2056 | 1.55E-05 | SNP3031 | 0.024575311 |
| SNP1015 | 0.007336571 | SNP2068 | 0.002251152 | SNP3035 | 0.000377076 |
| SNP1023 | 7.51E-27 | SNP2078 | 1.68E-07 | SNP3036 | 7.60E-08 |
| SNP1061 | 2.30E-16 | SNP2086 | 2.19E-18 | SNP3046 | 0.000584851 |
| SNP1066 | 0.046144784 | SNP2088 | 0.004965233 | SNP3056 | 0.026107664 |
| SNP1072 | 0.013264 | SNP2115 | 1.42E-129 | SNP3059 | 0.049868933 |
| SNP1074 | 0.02698823 | SNP2122 | 0.001489835 | SNP3065 | 0.007487225 |
| SNP1077 | 2.32E-06 | SNP2124 | 7.08E-14 | SNP3075 | 0.00013881 |
| SNP1089 | 0.010832619 | SNP2125 | 0.027563643 | SNP3076 | 3.63E-05 |
| SNP1093 | 0.039071123 | SNP2132 | 0.002251896 | SNP3086 | 0.000264645 |
| SNP1094 | 2.23E-08 | SNP2137 | 0.004250704 | SNP3092 | 0.001263167 |
| SNP1100 | 1.14E-08 | SNP2138 | 3.29E-13 | SNP3102 | 0.049712336 |
| SNP1106 | 0.032877202 | SNP2142 | 4.59E-22 | SNP3107 | 2.96E-10 |
| SNP1121 | 0.001380496 | SNP2149 | 1.38E-05 | SNP3118 | 0.006262825 |
| SNP1125 | 0.042121288 | SNP2153 | 8.36E-09 | SNP3125 | 6.61E-13 |
| SNP1129 | 0.006875284 | SNP2155 | 0.000976284 | SNP3131 | 0.003391636 |
| SNP1131 | 0.020616744 | SNP2162 | 0.013775033 | SNP3141 | 3.77E-11 |
| SNP1134 | 0.000568641 | SNP2169 | 0.024586495 | SNP3145 | 3.54E-21 |
| SNP1148 | 6.09E-11 | SNP2174 | 4.14E-08 | SNP3148 | 0.044113345 |
| SNP1162 | 0.000172619 | SNP2179 | 0.00358175 | SNP3154 | 0.005123067 |
| SNP1175 | 0.002288334 | SNP2183 | 3.62E-06 | SNP3184 | 0.015031746 |
| SNP1176 | 0.035817276 | SNP2189 | 6.34E-05 | SNP3185 | 0.002285186 |
| SNP1177 | 4.06E-11 | SNP2194 | 0.024168327 | SNP3195 | 4.38E-06 |
| SNP1202 | 0.010727942 | SNP2205 | 1.31E-08 | SNP3201 | 1.52E-05 |
| SNP1212 | 9.29E-07 | SNP2224 | 8.77E-12 | SNP3219 | 6.41E-08 |
| SNP1213 | 0.004628484 | SNP2225 | 1.52E-07 | SNP3226 | 0.000859762 |
| SNP1220 | 4.84E-09 | SNP2226 | 9.21E-12 | SNP3227 | 0.011477426 |
| SNP1226 | 3.46E-09 | SNP2237 | 9.58E-09 | SNP3256 | 1.15E-21 |
| SNP1235 | 0.013318264 | SNP2244 | 0.000596192 | SNP3257 | 4.45E-05 |
| SNP1240 | 0.007818405 | SNP2248 | 2.55E-12 | SNP3261 | 2.33E-06 |
| SNP1264 | 7.69E-05 | SNP2261 | 8.02E-15 | SNP3274 | 1.77E-06 |
| SNP1268 | 7.48E-09 | SNP2271 | 0.036437401 | SNP3288 | 1.58E-08 |
| SNP1274 | 0.001626846 | SNP2273 | 4.64E-07 | SNP3291 | 0.024168327 |
| SNP1278 | 5.96E-10 | SNP2274 | 1.70E-38 | SNP3318 | 1.29E-10 |
| SNP1294 | 4.80E-25 | SNP2275 | 2.56E-25 |  |  |
| SNP1313 | 0.011477426 | SNP2287 | 0.044113345 |  |  |

**Table S10** The loading value of outliers calculated using RDA.

| Axis | Snp_site | Loading | Bio2 | Bio5 | Bio9 | Bio12 | Bio15 | Bio17 | Predictor | Correlation |
| --- | --- | --- | --- | --- | --- | --- | --- | --- | --- | --- |
| 5 | SNP36 | 0.21413215 | 0.076063267 | 0.134504792 | 0.11660565 | 0.007010597 | -0.248612 | 0.231786698 | bio15 | 0.248611735 |
| 6 | SNP47 | -0.173911043 | -0.064513008 | 0.02301818 | -0.21804717 | -0.20189255 | -0.030113 | -0.176076992 | bio9 | 0.218047168 |
| 6 | SNP52 | 0.182984996 | -0.124298629 | 0.133752025 | 0.40105601 | 0.408645735 | -0.098085 | 0.354354533 | bio12 | 0.408645735 |
| 6 | SNP56 | -0.215838162 | -0.576929524 | 0.750735946 | 0.29544535 | 0.382628939 | -0.782608 | 0.504899746 | bio15 | 0.782608478 |
| 4 | SNP77 | -0.254207734 | -0.259696693 | 0.158526516 | -0.25963867 | -0.05443198 | -0.157999 | -0.0570551 | bio2 | 0.259696693 |
| 6 | SNP83 | 0.162978506 | 0.50619978 | -0.583532977 | -0.28294924 | -0.35457062 | 0.6168705 | -0.458764236 | bio15 | 0.616870504 |
| 4 | SNP128 | 0.232750134 | 0.09194137 | -0.058131098 | 0.1677865 | 0.109178075 | 0.0273054 | 0.071988009 | bio9 | 0.167786497 |
| 5 | SNP134 | 0.20508972 | 0.146489784 | -0.070268312 | -0.38671551 | -0.26832402 | -0.011043 | -0.173753249 | bio9 | 0.386715512 |
| 4 | SNP144 | 0.245949872 | 0.507485523 | 0.086616767 | -0.20517272 | -0.31484654 | -0.159879 | -0.174954118 | bio2 | 0.507485523 |
| 6 | SNP203 | 0.167678875 | -0.004665552 | 0.295616996 | 0.13893988 | 0.133527086 | -0.245957 | 0.166220852 | bio5 | 0.295616996 |
| 5 | SNP234 | -0.191099635 | 0.017629651 | 0.320896995 | 0.07816638 | 0.05841437 | -0.268799 | 0.05265844 | bio5 | 0.320896995 |
| 5 | SNP334 | 0.276695038 | 0.158572107 | -0.219692303 | 0.02126882 | -0.01594522 | 0.0808427 | 0.101350147 | bio5 | 0.219692303 |
| 5 | SNP345 | -0.188743438 | -0.004862144 | 0.273567073 | -0.00181097 | -0.0016016 | -0.236239 | -0.02110008 | bio5 | 0.273567073 |
| 6 | SNP404 | 0.167645132 | 0.03549283 | -0.120451921 | -0.04654276 | 0.052580523 | 0.1596284 | -0.040796769 | bio15 | 0.159628442 |
| 6 | SNP418 | 0.16281488 | -0.122961845 | 0.169390772 | 0.32239677 | 0.293199536 | -0.104999 | 0.262594865 | bio9 | 0.322396773 |
| 5 | SNP429 | 0.230239799 | -0.007292519 | -0.105187501 | -0.10363286 | -0.09160675 | 0.0147036 | 0.02225098 | bio5 | 0.105187501 |
| 4 | SNP441 | 0.242505468 | 0.313904574 | 0.291317165 | -0.09258486 | -0.21252112 | -0.365542 | -0.017626832 | bio15 | 0.365541561 |
| 4 | SNP443 | 0.268824598 | 0.055400414 | 0.091602978 | 0.20455022 | 0.105374135 | -0.132616 | 0.135911988 | bio9 | 0.204550216 |
| 4 | SNP464 | 0.24334805 | 0.403098507 | 0.089554573 | -0.15479594 | -0.27614519 | -0.162114 | -0.143000422 | bio2 | 0.403098507 |
| 5 | SNP470 | 0.214536808 | 0.069871691 | -0.038479719 | 0.01301282 | -0.06786936 | -0.061298 | 0.095138545 | bio17 | 0.095138545 |
| 5 | SNP486 | 0.212519087 | -0.204786199 | 0.068008168 | 0.38804533 | 0.346799706 | -0.159665 | 0.468623099 | bio17 | 0.468623099 |
| 4 | SNP531 | 0.243085644 | 0.383988186 | 0.151295613 | -0.16177921 | -0.27610193 | -0.222519 | -0.131440603 | bio2 | 0.383988186 |
| 4 | SNP586 | 0.237807106 | 0.110343443 | 0.23460512 | 0.22533694 | 0.130856526 | -0.311431 | 0.287740597 | bio15 | 0.311431285 |
| 5 | SNP615 | 0.218147717 | 0.139283315 | -0.178288517 | 0.140594 | 0.01242712 | 0.0826248 | 0.137873645 | bio5 | 0.178288517 |
| 5 | SNP682 | -0.213181915 | -0.40100668 | 0.185508137 | 0.45110649 | 0.515669095 | -0.10516 | 0.346500977 | bio12 | 0.515669095 |
| 6 | SNP709 | -0.170891932 | -0.023254589 | -0.001942289 | -0.19057739 | -0.09700614 | -0.082695 | -0.064109964 | bio9 | 0.190577388 |
| 4 | SNP869 | 0.230784825 | -0.003217628 | 0.32463425 | 0.20712971 | 0.179872576 | -0.381466 | 0.281049766 | bio15 | 0.381466188 |
| 5 | SNP897 | -0.197263582 | 0.02952845 | 0.305860525 | 0.13625506 | 0.087380782 | -0.230443 | 0.078639935 | bio5 | 0.305860525 |
| 5 | SNP991 | 0.234875528 | 0.058571978 | -0.349918808 | -0.01357439 | -0.00098738 | 0.2533392 | 0.020281832 | bio5 | 0.349918808 |
| 5 | SNP1134 | 0.252388421 | -0.00681015 | 0.182790704 | 0.05867435 | 0.027929186 | -0.294661 | 0.256350974 | bio15 | 0.29466079 |
| 5 | SNP1287 | 0.205542952 | 0.061804964 | -0.198974172 | 0.03118628 | -0.00250263 | 0.1283638 | 0.069909361 | bio5 | 0.198974172 |
| 5 | SNP1380 | 0.213900962 | -0.015086995 | -0.260296345 | -0.0338845 | -0.01501845 | 0.1825332 | 0.021592892 | bio5 | 0.260296345 |
| 6 | SNP1400 | 0.174840526 | 0.589987542 | 0.017248377 | -0.34435798 | -0.46276865 | -0.035827 | -0.30221471 | bio2 | 0.589987542 |
| 6 | SNP1407 | 0.178047714 | 0.100503794 | 0.017965939 | 0.01896388 | 0.005572077 | 0.0774362 | -0.069651719 | bio2 | 0.100503794 |
| 6 | SNP1505 | 0.162204771 | -0.035408831 | -0.289617921 | 0.2739796 | 0.235202049 | 0.3557184 | 0.088903852 | bio15 | 0.355718426 |
| 5 | SNP1645 | -0.20501913 | 0.084828988 | 0.004146374 | 0.11494058 | 0.115783517 | 0.071147 | -0.024050501 | bio12 | 0.115783517 |
| 4 | SNP1724 | -0.260013873 | -0.153401959 | -0.261956187 | -0.27861067 | -0.11489314 | 0.3168665 | -0.293821816 | bio15 | 0.316866499 |
| 5 | SNP1961 | 0.258172561 | 0.034657588 | -0.076198419 | 0.02421251 | -0.05642576 | -0.009626 | 0.115846054 | bio17 | 0.115846054 |
| 4 | SNP2018 | 0.229978029 | 0.399852297 | 0.146217023 | -0.19227744 | -0.26717716 | -0.25008 | -0.095651243 | bio2 | 0.399852297 |
| 4 | SNP2025 | 0.238808551 | -0.026788584 | 0.029174612 | 0.42658549 | 0.266634814 | -0.095678 | 0.368388925 | bio9 | 0.426585488 |
| 5 | SNP2037 | -0.200129028 | 0.204039161 | -0.070550193 | -0.02528163 | -0.07336547 | 0.1649762 | -0.190879942 | bio2 | 0.204039161 |
| 6 | SNP2046 | -0.164746224 | -0.261948799 | 0.403323904 | 0.03963309 | 0.107008876 | -0.475348 | 0.238245064 | bio15 | 0.475347751 |
| 5 | SNP2183 | -0.201857799 | -0.037376971 | 0.230356813 | -0.24332881 | -0.12801077 | -0.149847 | -0.202466218 | bio9 | 0.243328811 |
| 5 | SNP2188 | 0.216679254 | 0.114696915 | 0.148845383 | 0.17840375 | 0.087728498 | -0.252241 | 0.300159266 | bio17 | 0.300159266 |
| 6 | SNP2231 | 0.165092193 | 0.49470554 | -0.599912133 | -0.20137964 | -0.2261039 | 0.5886185 | -0.342592974 | bio5 | 0.599912133 |
| 4 | SNP2233 | -0.241440395 | -0.109635141 | 0.218235013 | -0.38755259 | -0.24080175 | -0.249065 | -0.134572748 | bio9 | 0.387552589 |
| 6 | SNP2272 | 0.218614747 | 0.081655755 | 0.041748784 | 0.13724645 | 0.103591977 | 0.0329439 | 0.061479088 | bio9 | 0.137246455 |
| 3 | SNP2322 | 0.323429883 | 0.012920686 | -0.120002316 | -0.10275749 | 0.112350463 | 0.0359925 | 0.05235772 | bio5 | 0.120002316 |
| 3 | SNP2242 | -0.180908813 | 0.028530999 | 0.349111289 | 0.20998538 | -2.60E-05 | -0.357913 | 0.188778294 | bio15 | 0.357912643 |
| 3 | SNP2471 | 0.301933202 | 0.065575286 | 0.021583894 | -0.10781181 | 0.053033633 | -0.059978 | 0.002710489 | bio9 | 0.107811809 |
| 6 | SNP2499 | 0.176934453 | 0.556045248 | -0.69946192 | -0.38560723 | -0.40644849 | 0.7106063 | -0.525579129 | bio15 | 0.710606331 |
| 6 | SNP2551 | 0.209150064 | -0.160263802 | 0.194214928 | 0.34681617 | 0.290742095 | -0.104301 | 0.280083492 | bio9 | 0.346816172 |
| 3 | SNP2588 | -0.250166288 | 0.202032089 | -0.467444284 | -0.05683204 | -0.16791937 | 0.4575176 | -0.217422361 | bio5 | 0.467444284 |
| 5 | SNP2611 | 0.205885722 | 0.013308216 | 0.147620372 | 0.12393944 | 0.076004162 | -0.233677 | 0.263469773 | bio17 | 0.263469773 |
| 5 | SNP2633 | 0.236800912 | 0.264736776 | -0.106560579 | -0.04791585 | -0.12932373 | -0.013609 | 0.036383363 | bio2 | 0.264736776 |
| 3 | SNP2660 | -0.278984781 | 0.111643878 | -0.166815137 | 0.13322832 | -0.05351399 | 0.1457779 | 0.000185818 | bio5 | 0.166815137 |
| 6 | SNP2669 | 0.211794377 | 0.06234451 | 0.119178634 | 0.15551168 | 0.193197397 | -0.100698 | 0.177993828 | bio12 | 0.193197397 |
| 5 | SNP2670 | -0.244086253 | 0.002856972 | 0.096979388 | 0.00452916 | 0.077119377 | -0.004453 | -0.079388408 | bio5 | 0.096979388 |
| 5 | SNP2699 | -0.195761752 | -0.143222866 | 0.286828983 | 0.05636339 | 0.130941877 | -0.226974 | 0.060610694 | bio5 | 0.286828983 |
| 5 | SNP2759 | 0.241010042 | 0.033136676 | -0.012149258 | -0.11435646 | -0.1606335 | -0.092325 | 0.025734197 | bio12 | 0.160633498 |
| 6 | SNP2769 | -0.163234009 | -0.021294211 | -0.021129612 | -0.25230174 | -0.12710309 | -0.026871 | -0.153240595 | bio9 | 0.252301743 |
| 4 | SNP2775 | 0.236902749 | 0.290649743 | 0.335594465 | -0.0660063 | -0.14251803 | -0.419044 | 0.055782835 | bio15 | 0.419043726 |
| 6 | SNP2778 | 0.176573983 | 0.001947795 | 0.269341806 | 0.16296127 | 0.133955031 | -0.198829 | 0.145713439 | bio5 | 0.269341806 |
| 6 | SNP2808 | 0.15977207 | 0.700054519 | -0.348895692 | -0.47239109 | -0.59829752 | 0.3723046 | -0.572705867 | bio2 | 0.700054519 |
| 3 | SNP2820 | -0.273271351 | 0.07669434 | -0.149837779 | 0.09054428 | -0.07498411 | 0.1343182 | -0.015300238 | bio5 | 0.149837779 |
| 6 | SNP2874 | 0.182826796 | 0.160697603 | -0.093671988 | 0.08591811 | -0.02949352 | 0.1320279 | 0.000491067 | bio2 | 0.160697603 |
| 6 | SNP2949 | 0.172751689 | 0.010650142 | 0.295999582 | 0.1006687 | 0.094562948 | -0.226616 | 0.112412318 | bio5 | 0.295999582 |
| 6 | SNP3031 | 0.171358285 | 0.481786926 | -0.78028602 | -0.2842705 | -0.2961923 | 0.8068207 | -0.482328669 | bio15 | 0.806820678 |
| 6 | SNP3034 | 0.17231467 | 0.073909942 | 0.365081106 | 0.29892303 | 0.164996732 | -0.31958 | 0.255074744 | bio5 | 0.365081106 |
| 6 | SNP3055 | 0.215589996 | 0.019419708 | 0.09079351 | 0.20083304 | 0.120248001 | 0.0211575 | 0.076420109 | bio9 | 0.200833038 |
| 6 | SNP3061 | 0.160393101 | -0.010792999 | 0.005254925 | -0.05697408 | 0.06589829 | 0.0461143 | -0.020281832 | bio12 | 0.06589829 |
| 6 | SNP3068 | 0.225040388 | 0.235616126 | -0.434690124 | -0.02588014 | -0.04285421 | 0.5311588 | -0.232054204 | bio15 | 0.531158781 |
| 3 | SNP3076 | 0.316736806 | -0.063262968 | 0.040350128 | -0.17950603 | 0.046810444 | -0.048161 | -0.05400117 | bio9 | 0.179506031 |
| 3 | SNP3109 | 0.314445209 | 0.005018583 | -0.138130247 | -0.04765686 | 0.154329012 | 0.0619255 | 0.081319905 | bio12 | 0.154329012 |
| 3 | SNP3112 | -0.237931159 | 0.048824462 | -0.351568776 | 0.10001219 | -0.00679827 | 0.3357263 | -0.037950109 | bio5 | 0.351568776 |
| 6 | SNP3119 | 0.180509964 | 0.045679945 | 0.454651257 | 0.17234044 | 0.112607137 | -0.397136 | 0.200256853 | bio5 | 0.454651257 |
| 5 | SNP3175 | 0.250305681 | -0.407255667 | -0.014269589 | 0.34349861 | 0.42965844 | -0.091291 | 0.481916419 | bio17 | 0.481916419 |
| 6 | SNP3220 | 0.177224883 | 0.065961511 | -0.007919594 | 0.07184815 | 0.093837505 | 0.0904015 | -0.012273673 | bio12 | 0.093837505 |
| 3 | SNP3269 | -0.204723012 | 0.004013799 | -0.018373783 | -0.23264878 | -0.28271269 | 0.0517548 | -0.260567228 | bio12 | 0.282712686 |
| 4 | SNP3279 | -0.252958619 | -0.440051106 | 0.086224764 | -0.05145686 | 0.119862138 | -0.087177 | 0.098552538 | bio2 | 0.440051106 |
| 5 | SNP3282 | -0.199190045 | -0.426724548 | 0.312049525 | 0.11122724 | 0.155426809 | -0.193558 | 0.091441657 | bio2 | 0.426724548 |
| 6 | SNP3283 | -0.188028543 | -0.099898016 | -0.230623821 | -0.34300524 | -0.22577855 | 0.2115697 | -0.286110184 | bio9 | 0.343005238 |
| 3 | SNP3296 | -0.233208999 | 0.073584459 | -0.304944558 | 0.01109866 | -0.08446495 | 0.2708465 | -0.092943289 | bio5 | 0.304944558 |
| 3 | SNP3304 | -0.199851705 | -0.030025338 | -0.086161024 | -0.16398404 | -0.19947539 | 0.0711875 | -0.174541398 | bio12 | 0.199475388 |
| 4 | SNP3339 | -0.250781741 | -0.129036115 | 0.026836049 | -0.28203757 | -0.1504383 | -0.017734 | -0.146107685 | bio9 | 0.282037574 |
| 3 | SNP3347 | -0.260972539 | -0.015525597 | -0.253064716 | 0.13496697 | 0.013314382 | 0.2405191 | 0.020727911 | bio5 | 0.253064716 |
| 3 | SNP3348 | -0.210271742 | 0.261736494 | -0.404068557 | -0.28586129 | -0.35074916 | 0.3725118 | -0.348084135 | bio5 | 0.404068557 |

**Table S11** The *p* value of outliers calculated using LFMM.

| Locus | Bio2 | Bio5 | Bio9 | Bio12 | Bio15 | Bio17 |
| --- | --- | --- | --- | --- | --- | --- |
| SNP72 | 0.955106476 | 0.02104004 | 0.544851396 | 0.075795974 | 0.021037909 | 0.00387506 |
| SNP255 | 0.106243778 | 0.92268197 | 0.175898904 | 0.998037506 | 0.998037506 | 0.998037506 |
| SNP282 | 0.055027683 | 0.999934179 | 0.998037506 | 0.998037506 | 0.999934179 | 0.999934179 |
| SNP290 | 0.716618287 | 0.146378356 | 0.993465705 | 0.07234138 | 0.113113991 | 0.030585781 |
| SNP354 | 0.047005147 | 0.999934179 | 0.642199905 | 0.547439057 | 0.999934179 | 0.999934179 |
| SNP358 | 0.024068247 | 0.998037506 | 0.882709753 | 0.813634638 | 0.999934179 | 0.999934179 |
| SNP404 | 0.07234138 | 0.350311095 | 0.010373778 | 0.642093054 | 0.583939315 | 0.96196567 |
| SNP443 | 0.999934179 | 0.091641409 | 0.011471351 | 0.998037506 | 0.062599059 | 0.099764474 |
| SNP490 | 0.015981852 | 0.999934179 | 0.782224424 | 0.75571311 | 0.999934179 | 0.999934179 |
| SNP644 | 0.001620674 | 0.998037506 | 0.25051036 | 0.998037506 | 0.998037506 | 0.998037506 |
| SNP651 | 0.053117918 | 0.998037506 | 0.279060131 | 0.356271617 | 0.998037506 | 0.999934179 |
| SNP718 | 0.703643277 | 0.993465705 | 0.072132433 | 0.998037506 | 0.998037506 | 0.83840812 |
| SNP740 | 2.19E-07 | 0.149592921 | 3.37E-06 | 0.062599059 | 0.569023974 | 0.955106476 |
| SNP763 | 0.872713833 | 0.955106476 | 0.072132433 | 0.998037506 | 0.983573278 | 0.981156961 |
| SNP895 | 0.052646648 | 0.998037506 | 0.726352039 | 0.998037506 | 0.999934179 | 0.998037506 |
| SNP1309 | 0.00387506 | 0.642093054 | 0.020554749 | 0.998037506 | 0.955106476 | 0.762086083 |
| SNP1406 | 0.998037506 | 0.027478467 | 0.913353562 | 0.028660909 | 0.05285308 | 0.015910568 |
| SNP1598 | 0.057917163 | 0.998037506 | 0.487678936 | 0.487678936 | 0.999934179 | 0.999934179 |
| SNP1634 | 0.047612015 | 0.829393394 | 0.072132433 | 0.999934179 | 0.969637554 | 0.564707277 |
| SNP1669 | 0.608114909 | 0.674639256 | 0.032008011 | 0.999934179 | 0.684542079 | 0.232955313 |
| SNP1777 | 0.350311095 | 0.984850965 | 0.047005147 | 0.969637554 | 0.998037506 | 0.919700407 |
| SNP1877 | 0.015981852 | 1.97E-11 | 0.000698448 | 1.15E-07 | 4.42E-12 | 9.91E-14 |
| SNP1981 | 0.116718231 | 0.998037506 | 0.642093054 | 0.998037506 | 0.999934179 | 0.998037506 |
| SNP1996 | 0.057917163 | 0.955106476 | 0.075795974 | 0.586045917 | 0.998037506 | 0.998037506 |
| SNP2031 | 0.00046233 | 0.998037506 | 0.998037506 | 0.375788428 | 0.998037506 | 0.999934179 |
| SNP2117 | 0.030585781 | 0.998037506 | 0.350311095 | 0.203834724 | 0.998037506 | 0.999934179 |
| SNP2129 | 0.05285308 | 0.988964323 | 0.331427469 | 0.998037506 | 0.998037506 | 0.881191633 |
| SNP2307 | 0.047114737 | 0.998037506 | 0.084015167 | 0.969637554 | 0.998037506 | 0.998037506 |
| SNP2322 | 0.108631955 | 0.998037506 | 0.047814965 | 0.279060131 | 0.998037506 | 0.998037506 |
| SNP2375 | 0.030585781 | 0.939476594 | 0.052646648 | 0.998037506 | 0.998037506 | 0.804577628 |
| SNP2435 | 0.097374722 | 0.117175456 | 2.03E-05 | 0.998037506 | 0.07234138 | 0.063714697 |
| SNP2578 | 0.747902822 | 0.017058418 | 0.392595619 | 0.047612015 | 0.015981852 | 0.00103659 |
| SNP2606 | 6.82E-05 | 0.999934179 | 0.921818604 | 0.136857339 | 0.998037506 | 0.999934179 |
| SNP2660 | 0.357421034 | 0.788061713 | 0.067469539 | 0.598883346 | 0.969637554 | 0.998037506 |
| SNP2669 | 0.0020743 | 0.487678936 | 0.060198594 | 0.448179803 | 0.83840812 | 0.998037506 |
| SNP2821 | 0.788061713 | 0.029384124 | 0.427302415 | 0.146378356 | 0.017695561 | 0.005723136 |
| SNP2947 | 0.016351068 | 0.959093268 | 0.047898418 | 0.998037506 | 0.998037506 | 0.866323427 |
| SNP2966 | 0.045012667 | 0.998037506 | 0.411878929 | 0.586045917 | 0.998037506 | 0.999934179 |
| SNP2969 | 4.46E-11 | 0.116718231 | 2.46E-08 | 0.517926289 | 0.415595902 | 0.07234138 |
| SNP3061 | 0.015634265 | 0.185097874 | 0.000698448 | 0.279060131 | 0.448067307 | 0.963820581 |
| SNP3096 | 0.075795974 | 0.782224424 | 0.080565301 | 0.998037506 | 0.969637554 | 0.88109884 |
| SNP3109 | 0.105793015 | 0.998037506 | 0.084015167 | 0.271018607 | 0.99912638 | 0.998037506 |
| SNP3119 | 0.053117918 | 0.586045917 | 0.703802766 | 0.993465705 | 0.969637554 | 0.998037506 |
| SNP3152 | 0.001412685 | 0.99912638 | 0.154563691 | 0.386158479 | 0.999934179 | 0.998037506 |
| SNP3171 | 0.079473028 | 0.998037506 | 0.998037506 | 0.998037506 | 0.998037506 | 0.999934179 |
| SNP3223 | 3.40E-10 | 0.999934179 | 0.331427469 | 0.018162724 | 0.998037506 | 0.999934179 |
| SNP3269 | 0.105793015 | 0.99858156 | 0.80538226 | 0.607313798 | 0.999934179 | 0.999934179 |
| SNP3304 | 0.076384308 | 0.998037506 | 0.544851396 | 0.716618287 | 0.998037506 | 0.998037506 |
| SNP3323 | 0.109487156 | 0.999934179 | 0.998037506 | 0.210855027 | 0.999934179 | 0.999934179 |

**Table S12** Summary of genetic variation associated with environment (env.), geography (geog.), and their combined effects in *Taiwania cryptomerioides*, based on redundancy analysis (RDA) using SNPs datasets with *K* = 5.

|  | All SNPs | Outlier SNPs | GEA SNPs | Putative selected SNPs |
| --- | --- | --- | --- | --- |
| Combined fractions |  |  |  |  |
| F~env. | 0.367*** | 0.480*** | 0.511*** | 0.474*** |
| F~geog. | 0.167*** | 0.239*** | 0.189*** | 0.230*** |
| Individual fractions |  |  |  |  |
| F~env. \| geog. | 0.279*** | 0.342*** | 0.470*** | 0.352*** |
| F~geog. \| env. | 0.095*** | 0.131*** | 0.203*** | 0.141*** |
| Total explained | 0.434*** | 0.532*** | 0.581*** | 0.530*** |
| Total confounded | 0.095 | 0.059 | - | 0.037 |
| Total unexplained | 0.566 | 0.468 | 0.419 | 0.47 |

Note: Data represent adjusted *R*^2^ values, and asterisks indicate statistical significance (**p* < 0.05, ***p* < 0.01, ****p* < 0.001). Total explained, total adjusted *R*^2^ of individual fractions. Total confounded, total of individual fractions confounded between combinations of climate and geography. F, dependent matrix of minor allele frequencies; RDA tests are of the form: F ~ independent matrices | covariate matrices. env., six retained environmental variables; geo., geography (longitude + latitude).
